# Assessment of the Safety and Potential Probiotic Properties of *Lactiplantibacillus plantarum* LP28 Based on Whole Genome Sequencing and Phenotypic and Oral Toxicity Analyses

**DOI:** 10.3390/microorganisms14040843

**Published:** 2026-04-09

**Authors:** Yi-Chu Liao, Yi-Chen Cheng, Chia-Chia Lee, Han-Yin Hsu, Yun-Fang Cheng, Shih-Hsuan Lin, Jin-Seng Lin, San-Land Young, Koichi Watanabe

**Affiliations:** 1 Culture Collection & Research Institute, SYNBIO TECH Inc., Kaohsiung 82151, Taiwan; yccheng@synbiotech.com.tw (Y.-C.C.); cclee@synbiotech.com.tw (C.-C.L.); hanin@synbiotech.com.tw (H.-Y.H.); fangcheng@synbiotech.com.tw (Y.-F.C.); shlin@synbiotech.com.tw (S.-H.L.); jslin@synbiotech.com.tw (J.-S.L.); s333@synbiotech.com.tw (S.-L.Y.); 2 Bioresource Collection and Research Center, Food Industry Research and Development Institute, Hsinchu 300193, Taiwan; 3 Department of Animal Science and Technology, National Taiwan University, Taipei 10673, Taiwan

**Keywords:** probiotics, *Lactiplantibacillus plantarum*, LP28, safety assessment, genotoxicity, oral toxicity, comparative genomic analysis, bacteriocins, whole-genome sequencing

## Abstract

*Lactiplantibacillus plantarum* LP28 (LP28), isolated from traditional Taiwanese dried tofu, has been demonstrated to have substantial probiotic potential because it increases the production of short-chain fatty acids (SCFAs) and strengthens anti-inflammatory responses. In this study, the safety of LP28 was assessed using both in vitro and in vivo approaches, including whole-genome sequence analysis, the Ames bacterial reverse mutation assay, a chromosomal aberration test, a rodent peripheral blood micronucleus test, a 28-day subacute oral toxicity assay, and an assessment of hemolytic activity. In vitro phenotypic evaluation revealed that LP28 exhibited no hemolytic activity and was susceptible to all the tested antibiotics except kanamycin. In vivo assessments revealed no significant alterations in reticulocyte counts or micronuclei incidence in ICR mice, and SD rats exhibited no subacute toxicity at an oral LP28 dosage of 2000 mg/kg body weight/day for 28 days. Moreover, a whole-genome sequence analysis of LP28 revealed the absence of antimicrobial resistance genes, harmful virulence factors, and genes associated with biogenic amine synthesis. Additionally, the presence of genes involved in stress responses (e.g., acid, bile salt, heat, osmotic, and oxidative stresses) and adhesion-related genes was confirmed. Furthermore, LP28 contains six genes (*plnA*, *plnE*, *plnF*, *plnJ*, *plnK,* and *plnN*) that encode bacteriocin precursor peptides, suggesting the potential for enhanced probiotic effects through the production of antimicrobial plantaricins. These findings highlight the potential of LP28 as a safe and effective probiotic for human consumption.

## 1. Introduction

Probiotics are live microorganisms, primarily bacteria, that provide health benefits, such as modulating the gut microbiota when they are consumed in sufficient amounts [1]. In recent decades, many probiotics have been sourced from intestinal or dairy product origins and utilized as starter cultures in the fermentation industry. However, with the growing awareness of lactose intolerance and milk allergies, plant-based fermented foods are receiving increasing attention as prospective alternatives [2,3]. In particular, these plant-based environments serve as excellent ecological niches that harbor novel lactic acid bacteria (LAB) with distinct metabolic adaptations. The natural microbial activity of these plant-associated microorganisms during fermentation or storage process offers benefits such as enhanced preservation and moderate flavor development. Consequently, the scientific interest has focused on exploring novel food-grade LAB strains with functional properties to further evaluate their potential as new probiotic candidates [4,5].

*Lactiplantibacillus plantarum* (*L. plantarum*) is naturally present in diverse environments, such as the gastrointestinal and vaginal tracts, plant materials, and various food products [6,7]. Owing to their health-promoting effects, a significant number of *L. plantarum* strains have been developed as probiotics [8,9,10,11,12]. The potential probiotic benefits of *L. plantarum* are varied and include its immunomodulatory, antioxidant [13,14], cholesterol-lowering [15,16], and antihypertensive [17] properties, as well as its ability to strengthen epithelial defense functions and maintain the gut microbial balance [18]. This species has been designated “qualified presumption of safety (QPS)” by the European Food Safety Authorities (EFSA) [19], and several strains of this species have been granted “generally recognized as safe (GRAS)” status by the United States Food and Drug Administration (FDA) (https://www.cfsanappsexternal.fda.gov/scripts/fdcc/?set=GRASNotices, accessed on 3 November 2025). Furthermore, there is a well-documented history of their safe consumption in fermented foods, supporting a wide range of applications [20,21]. However, because the functional properties of probiotics are strain-specific and probiotic products are designed for daily consumption, assessing the safety of probiotics is crucial for assessing potential interactions with the host and ensuring the safety of probiotic foods.

We previously demonstrated that the administration of *L*. *plantarum* LP28 (LP28), which is isolated from traditional Taiwanese dried tofu, enhances the production of short-chain fatty acids (SCFAs) and promotes the release of anti-inflammatory cytokines, ultimately preserving nerve fiber integrity and delaying the onset of neuropathic pain [22]. Consequently, a comprehensive evaluation of the safety and probiotic characteristics of LP28 is needed by precisely identifying genes associated with antimicrobial resistance, virulence factors [23], and biogenic amine synthesis [24] through whole-genome sequencing (WGS) analysis.

In this study, a combination approach integrating genomic analysis, in vitro tests, and in vivo experiments was used to assess the safety and potential probiotic properties of LP28 as a probiotic for human consumption. These included WGS analysis, an assessment of hemolytic activity, an antimicrobial susceptibility test, the Ames bacterial reverse mutation assay, a chromosomal aberration test, a rodent peripheral blood micronucleus assay, and a 28-day subacute oral toxicity analysis. These combined approaches provide a robust framework for assessing the potential of LP28 as a safe and effective probiotic for human consumption.

## 2. Materials and Methods

### 2.1. Bacterial Test Material

LP28 was isolated from “Dasi-dried tofu”, a traditional Taiwanese dried tofu obtained from a traditional market in Daxi, Taoyuan City, Taiwan. Briefly, the dried tofu sample was subjected to enrichment culture at 37 °C for 16–20 h. Samples were then serially diluted and inoculated on MRS agar plates. The plates were cultured under anaerobic conditions at 37 °C for 2–3 days. Distinct colonies with milk-coagulating ability were selected and screened by Gram staining and catalase testing. Gram-positive and catalase-negative isolates were subsequently identified based on 16S rRNA and *pheS* gene sequencing, and one of the isolates was designated as LP28.

For the current study, LP28 powder was prepared by SYNBIO TECH INC. (Kaohsiung, Taiwan). LP28 was cultured in MRS broth at 37 °C for 16 h, after which the cells were harvested via centrifugation. The resulting cell pellet was freeze-dried and kept at −20 °C for later applications. For the bacterial reverse mutation, chromosomal aberration, micronucleus, and 28-day subacute oral toxicity tests, LP28 powder was prepared by blending the freeze-dried bacterial cells with an appropriate quantity of maltodextrin to a concentration of 1 × 10^11^ CFU/g and stored at −20 °C until use.

### 2.2. Whole-Genome Sequencing and Taxonomic Identification

For whole-genome sequencing, LP28 genomic DNA was extracted as follows: the bacterial cell pellet was treated with lysozyme (50 mg/mL), achromopeptidase (30 mg/mL), proteinase K (50 mg/mL), 10% SDS, and RNaseA solution (100 mg/mL), at 37 °C for 1 h, 37 °C for 45 min, 55 °C for 1 h, 55 °C for 1 h, and 37 °C for 1 h, respectively. Subsequently, 1 mL of the mixture was mixed with protein precipitation solution (6 mL of 5 M potassium acetate, 1.15 mL of glacial acetic acid, and 2.85 mL of distilled water), followed by being kept on ice for 1 h. After centrifugation (14,000× *g* for 10 min, 4 °C), the supernatant was subjected to precipitation with 3 M sodium acetate (pH 5.4) and isopropanol. The DNA pellet was collected by centrifugation (12,000× *g* for 10 min, 4 °C), and washed with 70% ethanol, dried, and dissolved in 10 mM Tris-HCl (pH 8.5). The DNA was stored at −20 °C. The genome of LP28 was sequenced using an Oxford Nanopore Technologies Ligation Sequencing Kit (SQK-LSK109) on a MinION Flow Cell (R9.4.1) and using Illumina MiSeq in paired-end (2 × 150 bp) mode. After decoding and refinement, the validated data were hybrid assembled using Unicycler v0.4.9 software with Illumina high-accuracy short reads and ONT long reads to obtain the genome sequence of LP28.

The genome sequences of the reference bacterial strains were downloaded from the NCBI database. A phylogenomic tree based on whole-genome sequences was constructed using the TYGS web server (TYGS: https://tygs.dsmz.de/, accessed on 3 November 2025) [25]. The average nucleotide identity (ANI) values were determined using the JSpeciesWeb Server (JspeciesWS: https://jspecies.ribohost.com/jspeciesws/, accessed on 3 November 2025) [26], an online server for genome-based identification, and digital DNA–DNA hybridization (dDDH) values were obtained using the Genome-to-Genome Distance Calculator (GGDC) with the recommended Formula 2 [27].

### 2.3. Annotation and Comparative Analysis of Whole-Genome Sequences

Prokka software version 1.14.6 [28] was used to predict and annotate coding sequences (CDSs), transfer RNAs (tRNAs), ribosomal RNAs (rRNAs), and transfer–messenger RNAs (tmRNAs). The functional annotation of Cluster of Orthologous Groups (COG) categories was performed by querying the LP28 genome sequence against a locally installed eggNOG database version 5 [29] using the eggNOG-mapper tool version 2.1.12 [30]. Visualization of the circular map for the assembled LP28 genome was achieved through the online Proksee platform (Proksee: https://proksee.ca/, accessed on 18 August 2025). In addition, the LP28 genome was annotated using the RASTtk pipeline on the Rapid Annotation using Subsystem Technology (RAST) Prokaryotic Genome Annotation Server (RAST: http://rast.nmpdr.org/, accessed on 24 February 2025). Carbohydrate-Active Enzymes (CAZymes) analysis was conducted using the CAZy database [31]. Reference genomes for comparative genomics were downloaded from NCBI, specifically those of three *L. plantarum* strains: ATCC 14917^T^ (RefSeq assembly accession GCF_000143745.1), 299v (GCF_001888735.1), and WCFS1 (GCF_000203855.3). The GFF3 annotation files for these reference strains were produced with Prokka software and applied to pangenome evaluation using Roary version 3.11.2 [32]. OrthoVenn3 [33] facilitated the analysis of genomic orthologous clustering, incorporating the orthologous relationships among all predicted protein-coding genes.

### 2.4. Bioinformatics Analysis of LP28

Genes associated with virulence factors (VFs) and toxins were detected in the LP28 genome by cross-referencing the Virulence Factor Database (VFDB) [34], the Pathogenicity Island Database (PAIDB) [35], and the VirulenceFinder (https://cge.food.dtu.dk/services/VirulenceFinder/, accessed on 17 January 2025) and PathogenFinder (https://cge.food.dtu.dk/services/PathogenFinder/, accessed on 17 January 2025) datasets available through the Center for Genomic Epidemiology (CGE), to identify potential VFs, requiring >80% identity and >60% coverage. Protein sequences linked to microbial biogenic amine (BA) synthesis genes—such as histidine decarboxylase (EC 4.1.1.22), tyrosine decarboxylase (EC 4.1.1.25), ornithine decarboxylase (EC 4.1.1.17), lysine decarboxylase (EC 4.1.1.18), and agmatine deiminase (EC 3.5.3.12)—were sourced from UniProt (https://www.uniprot.org/, accessed on 17 January 2025) and assembled into a BLAST database (version 2.9.0+). Sequence data for probiotic-related genes from related strains were retrieved from the NCBI database and used to identify probiotic genes in *L. plantarum* strains. The BLASTp algorithm (version 2.9.0+) was employed to assess the BA production potential and probiotic functionality of LP28, using thresholds of >60% sequence identity, >70% query coverage, and an e-value < 1.0e–20.

Antimicrobial resistance (AMR) genes were investigated in the genome by consulting the Comprehensive Antibiotic Resistance Database (CARD) [36] with the Resistance Gene Identifier (RGI) software (version 6.0.5), utilizing DIAMOND homolog detection. The AMRFinder [37] and ResFinder (https://genepi.food.dtu.dk/resfinder, accessed on 17 January 2025) [38] databases were used to identify AMR genes according to their default settings. The Antibiotic Resistance Gene-Annotation (ARG-ANNOT) database [39] was searched for AMR genes using the BLASTn algorithm (version 2.9.0+), with parameters set at >80% identity and >60% coverage. The PHAge Search Tool (PHASTER: https://phaster.ca/, accessed on 22 August 2024) [40] and BAGEL4 (http://bagel4.molgenrug.nl/, accessed on 22 August 2024) [41] were utilized to detect phages and plasmids, respectively. Furthermore, the MobileElementFinder database v1.0.2 (https://cge.food.dtu.dk/services/MobileElementFinder/, accessed on 10 July 2025) [42] was used to identify mobile genetic elements (MGEs) and explore their potential links to antimicrobial resistance genes and virulence factors.

### 2.5. Antimicrobial Susceptibility Test

The minimal inhibitory concentrations (MICs) of LP28 were determined following the protocol outlined in ISO 10932:2010, in accordance with the European Food Safety Authority (EFSA) guidelines [43]. The susceptibility of LP28 to seven antibiotics—ampicillin, gentamicin, kanamycin, erythromycin, clindamycin, tetracycline, and chloramphenicol—was assessed using a broth microdilution method in 96-well plates. After inoculation, the plates were incubated anaerobically at 28 °C for 48 h. Bacterial growth was evaluated by measuring the optical density at 625 nm with a Sunrise Basic ELISA plate reader (Tecan, Grödig, Austria).

### 2.6. Bacterial Reverse Mutation Test (Ames Test)

Mutagenicity tests were conducted using five *Salmonella enterica* subsp. *enterica* serovar *Typhimurium* (*S. Typhimurium*) strains (TA98, TA100, TA102, TA1535, and TA1537) obtained from MOLTOX Inc. (Boone, NC, USA). The plate incorporation assay was performed in both the presence and absence of metabolic activation (S9 mix), according to the procedures outlined in the Organization for Economic Co-operation and Development (OECD) Guideline No. 471 [44]. Each bacterial strain was tested in seven groups: a negative control group (sterile water), a positive control group, and five LP28 treatment groups at concentrations of 5.000, 2.500, 1.250, 0.6250, and 0.3125 mg/plate. All groups were tested in triplicate. Briefly, 100 μL of an overnight bacterial culture, 100 μL of the corresponding test solution (negative control, positive control, or freshly prepared LP28 solution), and 0.5 mL of either 0.2 M phosphate buffer (pH 7.4) or phosphate buffer containing rat liver S9 mix were added to sterile tubes. The mixtures were gently agitated on a rotary shaker at 120 rpm and incubated at 36 ± 1 °C for 30 min. Subsequently, 2 mL of molten top agar (maintained at 48 ± 1 °C in a dry bath) supplemented with 200 μL of 0.5 mM histidine/biotin solution was added to each reaction tube. After mixing, the contents were poured onto minimal glucose agar plates. The plates were inverted and incubated at 36 ± 1 °C for 71 h. The number of spontaneous revertant colonies per plate was counted and compared with that of the negative control. A test sample was considered mutagenic if it met all of the following criteria: (i) the number of revertant colonies was significantly greater than that of the negative control (*p* < 0.05, one-way ANOVA with Dunnett’s post hoc test); (ii) the number of revertant colonies was at least twofold greater than that of the negative control; and (iii) a clear, dose-dependent increase in the number of revertant colonies was observed.

### 2.7. In Vitro Mammalian Cell Chromosomal Aberration Test

The Chinese hamster ovary (CHO)-K1 cell line was obtained from the Bioresource Collection and Research Center (BCRC, Hsinchu, Taiwan). To evaluate the cytotoxicity of LP28, a chromosomal aberration assay was conducted in vitro according to OECD Guideline No. 473 [45]. CHO-K1 cells were seeded in 6-cm dishes at a density of 1.5 × 10^6^ cells per dish and incubated for 24 h. LP28 powder was dissolved in culture medium and applied at concentrations of 2.0, 1.0, and 0.5 mg/mL. Cyclophosphamide monohydrate (CPP; 25 μg/mL) and mitomycin C (2.5 μg/mL) were used as positive controls under conditions with and without S9 metabolic activation, respectively. The negative control consisted of Ham’s F-12 medium supplemented with 10% fetal bovine serum. In the absence of the S9 mixture, the cells were exposed to the test substances for either short-term (3 h) or continuous (20 h) treatment. In the presence of the S9 mixture, only the short-term (3 h) exposure was conducted. Following treatment, the metaphase cells were harvested, fixed, and stained using the Diff–Quik method. For each treatment group, 100 metaphase cells were examined for chromosomal aberrations (CAs). A result was considered positive if it met all the following criteria: (i) the proportion of cells with chromosomal abnormalities in any of the LP28-treated groups was significantly greater than that in the negative control group (*p* < 0.05, Fisher’s exact test), and (ii) the proportion of cells with chromosomal abnormalities in the LP28-treated groups demonstrated a dose-dependent relationship. Data are expressed as the mean number of chromosomal aberrations observed in 300 metaphase cells in triplicate measurements.

### 2.8. Rodent Peripheral Blood Micronucleus Test

This study was conducted in accordance with OECD Guideline Test No. 474 [46]. All animal procedures were reviewed and approved by the Institutional Animal Care and Use Committee (IACUC) of Super Laboratory Co., Ltd. (New Taipei City, Taiwan) under approval number 113-11o. Twenty-five 6-week-old male ICR mice (BioLASCO Co., Ltd., Taipei, Taiwan) were housed under controlled environmental conditions with a 12-h light/dark cycle and randomly assigned to five groups (*n* = 5 per group): a negative control group (sterile water), a positive control group (cyclophosphamide monohydrate; CPP), and three LP28 treatment groups (500, 1000, and 2000 mg/kg BW). LP28 and sterile water were administered via oral gavage, respectively, while CPP was administered by intraperitoneal injection. All substances were administered twice at a 24-h interval. Peripheral blood samples (5 μL) were collected from the submandibular vein 46 h after administration. Whole blood was applied to acridine orange-coated slides and incubated in the dark at room temperature for 3 h. The proportion of reticulocytes (RETs) was determined per 2000 red blood cells (RETs/2000 RBCs), and the frequency of micronucleated reticulocytes (Mn-RETs) was evaluated per 4000 reticulocytes (Mn-RETs/4000 RETs) using a fluorescence microscope. A test result was considered positive if both of the following criteria were met: (i) the incidence of micronucleated reticulocytes in at least one LP28-treated group was significantly different from that of the negative control (*p* < 0.05, Kruskal–Wallis test with Dunn’s post hoc test), and (ii) the incidence of micronucleated reticulocytes increased in a dose-dependent manner across the LP28 treatment groups.

### 2.9. Repeated-Dose 28-Day Subacute Oral Toxicity Study in Rats

The study was conducted in accordance with OECD Guideline Test No. 407 [47]. Six-week-old male and female Sprague–Dawley (SD) rats were procured from BioLASCO Co., Ltd. The animals were randomly assigned to four groups, each comprising 12 rats (6 males and 6 females), and housed under controlled environmental conditions (12 h light/dark cycle, temperature maintained at 21 ± 1 °C, and relative humidity at 55 ± 10%). All animal procedures were reviewed and approved by the Institutional Animal Care and Use Committee (IACUC) of Super Laboratory Co., Ltd. (Approval No. 113-9i). LP28 was administered once daily via oral gavage at doses of 0, 500, 1000, or 2000 mg/kg body weight for a period of 28 consecutive days. Body weights were monitored and recorded weekly. Following an overnight fasting period, the animals were euthanized via carbon dioxide (CO_2_) inhalation. Major organs, including the testes or ovaries, adrenal glands, spleen, kidneys, heart, brain, and liver, were carefully excised, weighed, and subjected to histopathological examination. Blood samples were collected for hematological and serum biochemical analyses. Hematological parameters—comprising white blood cell (WBC) count, red blood cell (RBC) count, platelet count, neutrophil, lymphocyte, monocyte, eosinophil, and basophil counts, hemoglobin (Hb) concentration, hematocrit (Hct), mean corpuscular volume (MCV), mean corpuscular hemoglobin (MCH), and mean corpuscular hemoglobin concentration (MCHC)—were evaluated using an automated hematology analyzer (XT-1800i, Sysmex Corporation, Kobe, Japan). Prothrombin time (PT) was assessed using a CA-1500 Blood Coagulation Analyzer (Sysmex). Serum biochemical parameters, including glucose, blood urea nitrogen (BUN), creatinine, aspartate aminotransferase (AST), alanine aminotransferase (ALT), total protein, albumin, alkaline phosphatase (ALP), gamma-glutamyl transferase (γ-GT), total cholesterol, triglycerides, calcium, phosphorus, sodium, potassium, chloride, globulin, and total bilirubin, were measured using an automated clinical chemistry analyzer (7070 Autoanalyzer, Hitachi, Tokyo, Japan).

### 2.10. Hemolytic Activity

The hemolytic activity of LP28 was evaluated using MRS agar plates supplemented with 5% (*w*/*v*) defibrinated sheep blood (Creative Life Science, CMP0100311; Taipei, Taiwan). An overnight culture of LP28 was streaked onto the surface of the agar and incubated at 37 °C under anaerobic conditions for 48 h. α-Hemolytic *Streptococcus pneumoniae* ATCC 6305 [48] and β-hemolytic *Staphylococcus aureus* ATCC 25923 [49] were included as positive control strains. Hemolytic activity was determined by visual inspection of the zone surrounding the bacterial colonies. α-Hemolysis was indicated by a greenish halo, β-hemolysis by a clear, transparent halo, and γ-hemolysis by the absence of any discoloration or clearing (nonhemolytic activity).

### 2.11. Statistical Analysis

The results are presented as the mean ± SD. Statistical analyses were conducted using GraphPad Prism 7.04 (GraphPad Software, San Diego, CA, USA). For comparisons across multiple groups, the data were analyzed by one-way ANOVA with Tukey’s or Dunnett’s post hoc test (for the Ames test). The Kruskal–Wallis test with Dunn’s post hoc analysis was employed for multiple nonparametric comparisons. A *p* value less than 0.05 was considered to indicate statistical significance.

## 3. Results

### 3.1. Identification of LP28

The first step in the safety assessment is the accurate identification of the probiotic strain at the species level. Phylogenetic analysis based on 16S rRNA gene sequences indicated that strain LP28 belonged to the genus *Lactiplantibacillus* and shared high sequence similarity (>99.8%) with the type strains of its closest phylogenetic neighbors, including *L. pentosus* (100%) and *L. plantarum* (99.9%), as well as *L. argentoratensis* and *L. paraplantarum* (99.8%) (Figure S1, Table S1). Phylogenetic analysis based on the *pheS* gene sequences further revealed that strain LP28 clustered with *L. plantarum* JCM 1149^T^ (=CIP 103151^T^), showing 100% sequence similarity (Figure S2, Table S1). Subsequent phylogenomic analysis of whole-genome sequences between LP28 and its ten closely related species in the genus *Lactiplantibacillus* revealed that LP28 and *L. plantarum* ATCC 14917^T^ formed an independent cluster (Figure S3). The average nucleotide identity (ANI) values between LP28 and each of its closest species, *L. plantarum*, *L. argentoratensis*, and *L. paraplantarum*, were 99.07%, 94.82%, and 85.43%, respectively; the digital DNA–DNA hybridization (dDDH) values were 94.1%, 62.6%, and 31.6%, respectively (Table S2). ANI and dDDH values of 95–96% and 70%, respectively, are the most widely accepted thresholds for species delineation. Therefore, based on these genotypic results, LP28 was identified as *Lactiplantibacillus plantarum*.

### 3.2. Genome Structure and General Features of LP28

**Figure 1:**
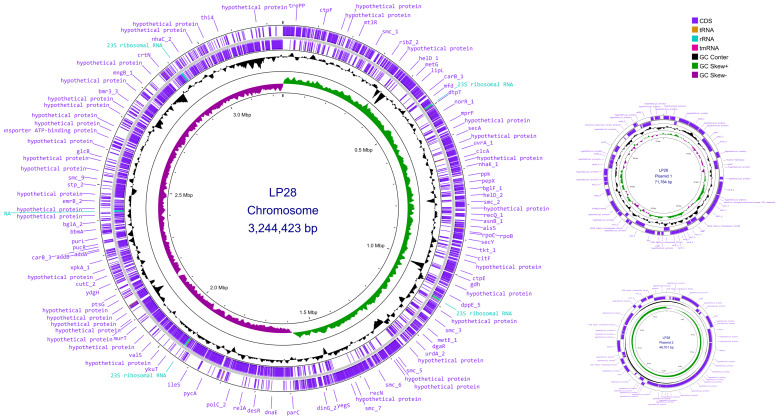
Circular visualization of the LP28 genome. From the outer to inner layers: the first and second circles represent forward and reverse CDSs (coding sequences) annotated using Prokka, respectively, including tRNA, rRNA, and tmRNA; the third circle indicates the GC content; the fourth circle depicts the GC skew (G − C)/(G + C); and the fifth circle represents the genome size. The genome of LP28 comprises three contigs: a circular chromosome of 3,244,423 bp, and two plasmids (plasmid 1: 71,784 bp, and plasmid 2: 48,701 bp).

Whole-genome sequencing of LP28 was conducted using both the Illumina MiSeq platform and the Oxford Nanopore MinION platform. As shown in Figure 1, the genome of LP28 comprises three contigs: a circular chromosome of 3,244,423 bp, and two plasmids (plasmid 1: 71,784 bp, and plasmid 2: 48,701 bp). The size of the whole-genome sequence of LP28 was 3,364,908 bp. Genomic annotation using Prokka predicted a total of 3299 genes, including 3144 protein-coding sequences, 16 rRNAs, 68 tRNAs, and one tmRNA (Table S3). The circular genomic map, which includes highlighted key genomic characteristics such as gene distribution on the forward and antisense strands, functional classification of genes, and GC content, is shown in Figure 1.

### 3.3. Functional Annotation of the Genome

Functional annotation based on the RAST showed that LP28 genes were classified into 23 feature types and 821 subsystems according to SEED subsystem categorization. Among these subsystems, “Carbohydrates” was the most represented subsystem (147 genes), followed by “Amino Acids and their derivatives” (122 genes) and “Protein Metabolism” (105 genes). The differences in the SEED subsystem features between LP28 and the reference strains (ATCC 14917^T^, 299v, and ACFS1) are shown in Table S4. According to the COG annotation, the 2823 coding sequences (CDSs) of the LP28 genome were classified into 19 functional categories. To focus on features relevant to its metabolic adaptation, a significant proportion of genes were classified in the COG categories “Carbohydrate Transport and Metabolism” (G: 269 genes, 9.5%) and “Amino Acid Transport and Metabolism” (E: 225 genes, 8.0%) (Table S5). Comparative analysis of the LP28 genome with those of ATCC 14917^T^, 299v, and WCFS1 using SEED subsystems and COG categories revealed that LP28 shares a highly similar functional profile with these reference strains. The CAZyme distribution across LP28, ATCC 14917^T^, 299v, and WCFS1 strains revealed a high degree of conservation with notable subtle variations. The LP28 genome comprises 206 genes within the CAZyme gene families, distributed as follows: 115 glycoside hydrolases (GHs), 70 glycosyltransferases (GTs), two carbohydrate esterases (CEs), three auxiliary activity (AA) enzymes, and 16 carbohydrate-binding modules (CBMs). LP28 exhibited high levels of CBM50 (LysM domains), GH1 (*β*-glucosidase), GH13 (*α*-amylase), GT2 (various glycosyltransferases), and GT4 (*α*-glucosyltransferase), which are consistent with the levels observed for ATCC 14917^T^, 299v, and WCFS1. Additionally, GH8 (endoglucanase) was exclusively predicted in the LP28 strain. The CAZyme profile of LP28 shows high similarity to probiotics such as strains 299v and WCFS1, with only slight differences. This finding indicates that LP28 possesses metabolic capabilities adapted to complex carbohydrate degradation, suggesting potential for probiotic application similar to 299v and WCFS1 (Figure S4).

### 3.4. Comparative Genomic Analysis

**Figure 2:**
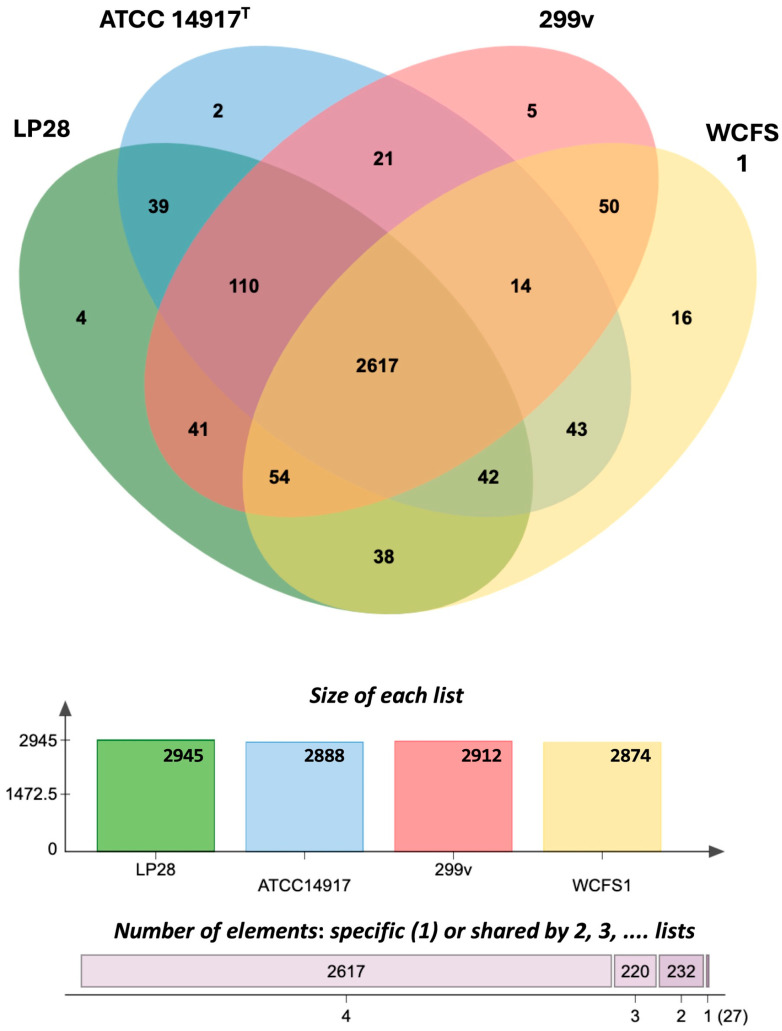
Comparative genome analysis. Venn diagram showing the distribution of shared gene families (orthologous clusters) among LP28, ATCC 14917^T^, 299v, and WCFS1. Totals of orthologs in each genome were used to generate the Venn diagram. A total of 2617 orthologous clusters were shared among all four strains, 220 were shared among three strains, and 232 were shared among two strains; 27 strain-specific singleton clusters were identified in LP28 (4), ATCC 14917^T^ (2), 299v (5), and WCFS1 (16).

The OrthoVenn3 web server was used to assemble an *L. plantarum* pangenome using the four genomes of LP28, ATCC 14917^T^, 299v, and WCFS1. The clinically documented commercial probiotics 299v and WCFS1 were selected to serve as ‘gold-standard’ baselines for evaluating LP28. The strain ATCC 14917^T^, the type strain of *L. plantarum*, was also included in this analysis. Among the clusters, a total of 2617 shared clusters of orthologous groups were identified across all four strains, 220 among the three strains, and 232 among the two strains. Furthermore, the genomes of LP28, ATCC 14917^T^, 299v, and WCFS1 each contain 4, 2, 5, and 16 unique protein clusters, respectively (Figure 2). A subsequent functional analysis of the four clusters specific to LP28 revealed that only one of these clusters had a predicted role, encoding transmembrane transporter activity (GO:0022857), whereas the other three clusters (cluster2845, cluster2846, and cluster2848) remained unannotated (lacking Swiss-Prot Hits or Gene Ontology Annotations), as outlined in Table S6. Among the 16 unique protein clusters found in WCFS1, three protein clusters were associated with DNA packaging (GO:0006323), NADPH regeneration (GO:0006740), and carbohydrate:proton symporter activity (GO:0005351), whereas the remaining 13 clusters were unannotated. Similarly, among the five unique protein clusters found in 299v, two clusters were associated with the plasma membrane (GO:0005886) and transmembrane transport (GO:0055085), whereas the remaining three clusters were unannotated. The limited functional annotation suggests the novelty of these clusters. These findings indicate that, despite the high ANI values among the four *L. plantarum* strains (98.76–99.27%), each genome harbors distinct features and metabolite biosynthesis potential, likely resulting from adaptations to specific ecological niches.

### 3.5. Comparative Analysis of Bacteriocin-Producing Gene Clusters from Different Strains

**Figure 3:**
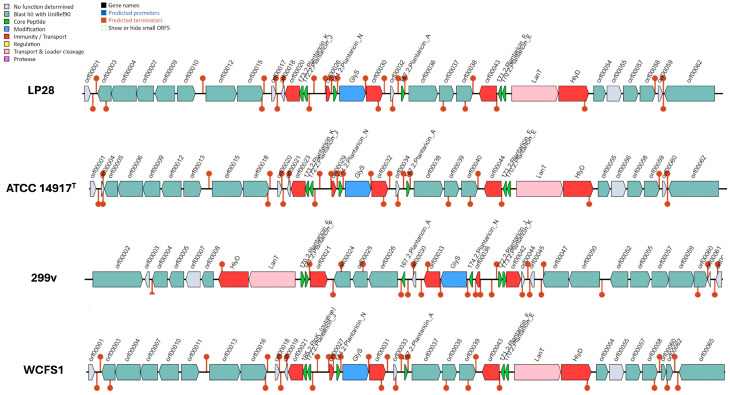
Comparative analysis of bacteriocin-producing gene clusters in *Lactiplantibacillus plantarum* strains. The identified genes included *plnA* (167.2, plantaricin A), *plnE* (170.2, plantaricin E), *plnF* (171.2, plantaricin F), *plnJ* (172.2, plantaricin J), *plnK* (173.2, plantaricin K), *plnN* (174.2, plantaricin N), LanT (bacteriocin ABC-transporter), and HlyD (accessory factor for ABC-transporter PlnH). Color coding: red blocks, immunity and transport genes; green arrows, core peptide genes; pink blocks, transport and leader cleavage genes; blue blocks, peptide modification genes; gray blocks, genes with undetermined function.

Bacteriocins are antimicrobial peptides that are commonly produced by food-grade LAB and have been extensively studied for their potential as inherent food preservatives due to their antimicrobial effects. Specifically, many *L. plantarum* strains have been reported to produce plantaricins, a distinct taxonomic class of bacteriocins [50,51]. The use of BAGEL4 for whole-genome analysis of LP28 revealed the presence of six genes encoding bacteriocin precursor peptides, namely, *plnA*, *plnN*, *plnEF* (two-peptide plantaricin), and *plnJK* (two-peptide plantaricin), which are responsible for the production of bacteriocin precursors. As illustrated in Figure 3, the *pln* gene clusters identified in LP28, ATCC 14917^T^, 299v, and WCFS1 shared highly conserved synteny, defined as gene order. Specifically, they all possess the essential genetic architecture for producing class IIb two-peptide bacteriocins, along with the relevant transporters (LanT/HlyD) and immunity genes. This finding indicates that strains LP28, ATCC 14917^T^, 299v, and WCFS1 possess a similar functional capacity to produce plantaricins.

### 3.6. Analysis of Probiotic-Related Genes

To evaluate the probiotic potential of LP28 at the genomic level, we analyzed genes associated with stress resistance and cell adherence in LP28 and three reference strains (ATCC14917^T^, 299v, and WCFS1), as summarized in Table 1. The LP28 genome harbored the gene LP28_02863 encoding cholylglycine hydrolase for bile salt tolerance and eight genes (LP28_01986 to LP28_01993) encoding F_0_F_1_ ATP synthase for acid tolerance. The following genes were identified as encoding chaperone proteins involved in the heat stress response: ClpB (LP28_01581), DnaJ (LP28_01668), DnaK (LP28_01669), 33 kDa chaperonin (LP28_00458), GroES (LP28_00562), and GroEL (LP28_00563). In addition, four genes encoding carnitine transport proteins for osmotic stress adaptation, OpuCA (LP28_01329), OpuCB (LP28_01330), OpuCC (LP28_01331), and OpuCD (LP28_01332), and the genes for oxidative stress resistance, encoding proteins such as thiol peroxidase (LP28_01947), thioredoxin reductase (LP28_00597), and glutaredoxin (LP28_00535), which catalyze glutathione-dependent disulfide reduction [52], were identified in the LP28 genome. These genes were conserved across LP28, ATCC14917^T^, 299v, and WCFS1, reflecting a shared genetic capability for stress resistance among the strains. The major adhesion protein in Gram-positive bacteria is sortase, which was encoded by the genes *srtA* and *sasA* identified in the genome of strain LP28. The *srtA* gene (LP28_00422), encoding an LPXTG-specific sortase, was also detected in strain 299v (299v_01910) but not in strains ATCC14917^T^ or WCFS1. Additionally, four genes encoding SasA proteins with an LPXTG motif (LP28_02089, LP28_01362, LP28_02609, LP28_02562) were identified in LP28. The *eno* genes (LP28_00624 and LP28_01596), which encode enolases associated with the binding of adhesion [53], were detected in LP28. The *fbaA* gene encoding fructose-bisphosphate aldolase (LP28_00283) and *scp* genes encoding segregation and condensation proteins A (LP28_01572) and B (LP28_01571) were present in LP28.

### 3.7. Safety Evaluation of LP28

#### 3.7.1. Analysis of Genotoxicity

The genotoxic potential of LP28 was evaluated using the Ames test, a chromosomal aberration assay, and a peripheral blood micronucleus assay. Mutagenicity was assessed through a bacterial reverse mutation assay employing five *S. Typhimurium* strains (TA98, TA100, TA102, TA1535, and TA1537), which were exposed to various concentrations of LP28. Compared with the negative control group, LP28 treatment at different concentrations did not induce a twofold or greater increase in the mean number of revertant colonies in TA98, TA1535, or TA1537, regardless of the presence or absence of the S9 metabolic activation system. No statistically significant differences were observed (*p* > 0.05), and no dose-dependent effects were noted. In the TA100 strain, compared with the negative control treatment, treatment with 0.3125 mg of LP28 resulted in a significant increase in the number of revertant colonies in both the presence and absence of S9. In the TA102 strain, a significant increase in the number of revertant colonies compared with that in the negative control was observed following LP28 treatments of 2.5, 1.25, and 0.3125 mg with the S9 mix. However, this increase did not exceed the required twofold threshold. As anticipated, the positive controls exhibited significant increases in the number of revertant colonies, both with and without S9 activation (*p* < 0.0001) (Table 2). These results demonstrated that LP28 does not display mutagenic activity toward histidine auxotrophic *S. Typhimurium* strains.

In the chromosomal aberration assay, CHO-K1 cells were treated with LP28 at concentrations of 0.5, 1.0, and 2.0 mg/mL, after which the frequency of cells displaying CAs was evaluated. In the positive control groups, treatment with CPP in the presence of S9 metabolic activation or mitomycin C without S9 resulted in CA frequencies ranging from 12.7 to 13.3%. In the absence of S9 activation, CHO-K1 cells exposed to LP28 for 3 or 20 h presented CA frequencies between 1.3% and 2.3%. Similarly, with S9 activation after 3 h of exposure, the CA frequency in LP28-treated cells remained within the same range of 1.3–2.3%. In contrast, the positive control under these conditions presented a markedly higher CA frequency (13.3%). The CA frequencies of the negative control groups ranged from 0.7 to 2.3% (Table 3). These results indicate that LP28 did not significantly increase chromosomal aberrations under any tested condition, suggesting that it is not genotoxic to CHO-K1 cells at concentrations up to 2.0 mg/mL.

The genotoxic potential of LP28 was further evaluated in vivo using a peripheral blood micronucleus assay in ICR mice. Mice were orally administered LP28 at doses of 500, 1000, and 2000 mg/kg body weight, and after 46 h, the percentage of reticulocytes among total red blood cells and the frequency of micronucleated reticulocytes (MN-RETs) were measured. In the CPP-treated positive control group, the reticulocyte ratio (RETs per 2000 RBCs) was 17.6 ± 2.8%, which was significantly lower than that in the negative control group, indicating bone marrow suppression. Additionally, the frequency of MN-RETs (per 4000 RETs) in the positive control group was substantially greater than those in both the negative control and LP28-treated groups. In contrast, compared with the negative control, LP28 at all the tested doses did not significantly alter the reticulocyte ratio or the incidence of micronucleated reticulocytes (Table 4). These results indicated that LP28 lacks genotoxic effects in the in vivo peripheral blood micronucleus assay in mice.

#### 3.7.2. Repeated-Dose 28-Day Subacute Oral Toxicity Study in Rats

LP28 was administered orally to SD rats once daily at doses of 500, 1000, and 2000 mg/kg body weight for 28 consecutive days. No abnormal clinical signs or changes in general behavior or physical activity were observed in either the control or treatment groups throughout the study period. Although the body weight of each group increased, no significant differences were observed between the LP28-treated and control groups in either male or female rats. At the end of the 28-day treatment period, all the animals were euthanized, and major organs, including the testes/ovaries, adrenal glands, spleen, kidneys, heart, brain, and liver, were collected for weight measurement and histopathological examination. In male rats, the adrenal gland weights in both the low- and high-dose LP28 groups were significantly greater (*p* < 0.05) than that in the control group. However, no dose-dependent trend was observed, and histological analysis revealed no treatment-related lesions. Therefore, these findings were not considered toxicologically relevant. In female rats, no significant differences in organ weight were observed between the LP28-treated groups and the control group (Table 5). Moreover, no mortality or treatment-related changes in appearance or behavior were noted during the 28-day study period.

After 28 consecutive days of oral LP28 administration, blood samples were collected from all the rats for hematological and serum biochemical analyses. LP28 treatment did not result in significant alterations in most of the hematological parameters evaluated. In female rats, the eosinophil count was significantly greater (*p* < 0.05) in the low-dose group than in the high-dose group (Table S7). Additionally, compared with those in the control group, triglyceride and potassium levels in the high-dose group were significantly lower (*p* < 0.05). In female rats, total protein and albumin levels were significantly lower in the high-dose group than in the control group (*p* < 0.05), whereas potassium levels were significantly higher in the medium-dose group than in the high-dose group (*p* < 0.05) (Table S8). Despite these statistically significant differences, no dose-dependent trends were observed, and all the measured values remained within the established physiological reference ranges. Therefore, these changes were not considered to be biologically significant or related to LP28 treatment.

#### 3.7.3. Hemolytic Activity

Blood agar, which is composed of general nutrient medium supplemented with 5% (*w*/*v*) defibrinated sheep blood, is widely used to evaluate the hemolytic potential of bacterial strains. In the present study, LP28, along with two reference strains, *S. pneumoniae* ATCC 6305 and *S. aureus* ATCC 25923, was streaked onto sheep blood agar plates and incubated at 37 °C for 48 h under anaerobic conditions to assess hemolytic activity. As shown in Figure S5, LP28 did not induce any visible hemolysis on the agar surface, indicating a γ-hemolytic phenotype (nonhemolytic). In contrast, the hemolytic patterns of the control strains were distinct: *S. pneumoniae* ATCC 6305 produced a greenish halo characteristic of α-hemolysis, whereas *S. aureus* ATCC 25923 formed a clear, transparent zone indicating β-hemolysis.

#### 3.7.4. Antimicrobial Resistance and Associated Genes

The antimicrobial susceptibility profile of LP28 was assessed by determining the minimum inhibitory concentrations (MICs) for seven antibiotics: ampicillin, gentamicin, kanamycin, erythromycin, clindamycin, tetracycline, and chloramphenicol. These MIC values were compared against the microbiological cutoff values established by the European Food Safety Authority (EFSA) for *L. plantarum/pentosus*. As summarized in Table 6, LP28 was susceptible to all the tested antibiotics except kanamycin. The MIC for kanamycin was 128 mg/L, which exceeded the EFSA cutoff value by twofold, indicating resistance. When a bacterial strain is resistant to a specific antibiotic, identification of the underlying antimicrobial resistance (AMR) determinants is critical [42]. To address this, we conducted a genome-wide search for AMR genes in the LP28 genome using the CARD, AMRFinder, ResFinder, and ARG-ANNOT databases. Additionally, the potential for gene transfer was evaluated using the Mobile Element Finder and PHASTER databases. No genes associated with resistance to ampicillin, gentamicin, kanamycin, erythromycin, clindamycin, tetracycline, or chloramphenicol were detected in the LP28 genome (Table S9). In the mobile element analysis, 16 regions were identified—five ISP2, six ISP1, two ISLsa1, and three ISLpl1 regions—all of which were classified as insertion sequences (Table S10). Furthermore, phage analysis revealed three intact phage regions (Table S11). However, no antibiotic resistance genes were detected within these phage regions or mobile element sequences, suggesting that there is no risk of horizontal gene transfer (HGT) of AMR genes from LP28 to other bacteria.

#### 3.7.5. Determination of Virulence Factors and Toxin-Related Genes

VFs refer to elements (i.e., gene products) that allow a microorganism to colonize a host niche, proliferate, and cause tissue damage or systemic inflammation. VFs include secreted proteins such as protein toxins and enzymes, as well as cell-surface structures such as capsular polysaccharides, lipopolysaccharides, and outer-membrane proteins that directly contribute to disease processes [34,54]. To identify known VFs and toxin-related genes and hence the determinants of pathogenicity, all the annotated genes of LP28 were analyzed using BLASTn and the VFDB, PAIDB, and CGE databases. No putative virulence genes were identified under the stringent criteria of >80% sequence identity and >60% query coverage.

#### 3.7.6. Biogenic Amine (BA)-Producing Genes

The role of BAs in terms of both food spoilage and food safety is a significant concern. The production of BAs is catalyzed mainly by the microbial-mediated decarboxylation of amino acids, particularly tyrosine, histidine, and tryptophan, into tyramine, histamine, and tryptamine, respectively, and some *Lactobacillus* strains produce biogenic amines via amino acid decarboxylase activity [55,56,57,58]. A search of the LP28 genome for genes related to adverse metabolite production revealed no genes associated with the production of BAs, including genes coding for the enzymes histidine decarboxylase (EC 4.1.1.22), tyrosine decarboxylase (EC 4.1.1.25), ornithine decarboxylase (EC 4.1.1.17), lysine decarboxylase (EC 4.1.1.18), and agmatine deiminase (EC 3.5.3.12), which are responsible for the synthesis of BAs in LAB [59].

## 4. Discussion

In the present study, a comparative genomic analysis of the *Lactiplantibacillus plantarum* LP28 strain was conducted to elucidate its safety and probiotic potential. The LP28 genome comprises a 3,244,423 bp circular chromosome and two plasmids (71,784 bp and 48,901 bp), with a GC content of 44.3%. Among the 3144 protein-coding genes identified, 2823 were functionally annotated. Subsequent functional analysis revealed a dominance of carbohydrate metabolism genes (147 genes) and a significant proportion of genes with unknown functions (20.5% in COG classification), reflecting the metabolic versatility of *L. plantarum*. The CAZyme profile revealed 206 genes, including a large number of genes involved in CBM50 (carbohydrate-binding modules), GH1 (*β*-glucosidase; EC 3.2.1.21), GH13 (*α*-amylase; EC 3.2.1.1), GH36 (*α*-galactosidase; EC 3.2.1.22), GT2 (various glycosyltransferases), and GT4 (*α*-glucosyltransferase; EC 2.4.26), along with a unique GH8 (endoglucanase; EC 3.2.1.4) gene. These CAZymes, particularly GHs, catalyze the synthesis and decomposition of complex carbohydrates by hydrolyzing glycosidic bonds, playing crucial roles in polysaccharide degradation, gut carbohydrate metabolism, and fermented food flavor development [60,61,62]. The high similarity of LP28 with reference strains (ATCC 14917^T^, 299v, and WCFS1) provides a robust genomic baseline for determining its safety and probiotic properties. Furthermore, the presence of these enzymes in LP28 indicates specialized cellulose degradation capabilities that complement α-galactosidase and α-amylase activities, enabling comprehensive dietary fiber metabolism and enhanced carbohydrate utilization in the gut environment.

Bacteriocins, a large family of ribosomally synthesized antimicrobial peptides produced by numerous food-grade LAB, have attracted significant attention because of their ability to inhibit the growth of various undesirable microorganisms. This potent antimicrobial effect makes them attractive as inherent and natural preservatives for food applications, offering a cleaner alternative to synthetic chemical additives [63,64]. The findings of this study are consistent with substantial prior research regarding the high productivity of *L. plantarum* as a bacteriocin producer. The ability of a significant number of L. plantarum strains to produce plantaricins, a distinct subclass of bacteriocins, has been well documented in the literature [50,51,65]. For instance, *L. plantarum* MKTJ24, which possesses plantaricin gene clusters, has been shown to upregulate mucin genes and decrease LPS-induced reactive oxygen species (ROS) and nitric oxide (NO) production in cell lines [66]. Furthermore, *L. plantarum* ULAG24, harboring plantaricin genes, exhibited antagonism against foodborne pathogens and stimulated IFNγ and IL10 in mice [67]. In addition, an in vivo study further demonstrated that the bacteriocin-producing *L. plantarum* strains Q7 and F3-2 upregulate the gene expression of intestinal tight junction proteins in mice, improve the gut microbial structure and promote short-chain fatty acid synthesis in mice [68]. LP28 was also identified as a bacteriocin-producing strain using BAGEL4, as evidenced by the presence of the *plnA*, *plnE*, *plnF*, *plnJ*, *plnK*, and *plnN* gene clusters; all these genes were proven to have strong antibacterial properties. The conservation of these bacteriocin genes across the reference strains (ATCC 14917^T^, 299v, and WCFS1) suggests that LP28 has a potential competitive advantage in inhibiting pathogenic bacteria, consequently enhancing its probiotic potential. These findings demonstrate the dual applicability of LP28 in food safety preservation and gut microbiota modulation, suggesting its high potential as a promising probiotic candidate.

LAB are widely used in food fermentation due to their remarkable ability to resist diverse environmental stresses, including low pH, high salinity, and temperature variations [69]. Their ability to survive in varied conditions is a distinctive feature that demonstrates their utility in industrial and artisanal applications. Stress-adaptive proteins play key roles by regulating tolerance mechanisms and driving genetic changes that improve survival under harsh conditions. As summarized in Table 1, the various stress resistance genes detected in the LP28 genome suggest that this strain can survive under industrial stress conditions and in the gastrointestinal environment [70,71]. The LP28 strain contains genes encoding key heat shock proteins, such as DnaJ, DnaK, GroES, and GroEL, along with ClpB for protein refolding under thermal stress. These molecular chaperones, which are essential for industrial fermentation tolerance, work alongside the F_0_F_1_ ATP synthase gene cluster to maintain proton gradients and pH homeostasis during acid stress from lactic acid accumulation. Additionally, the gene encoding cholylglycine hydrolase was present, indicating that LP28 can deconjugate bile salts—a key ability for surviving intestinal bile acids [72]. According to previous studies, the Na+/H+ antiporter system, complemented by ATP synthase, pumps protons out using cellular ATP to maintain intracellular pH homeostasis [64]. In this study, the genes in LP28 encoding carnitine transport proteins (OpuCA, OpuCB, OpuCC, and OpuCD) associated with osmotic stress adaptation were also identified. These findings indicate that strain LP28 possesses genetic features that facilitate tolerance to gastric acid, bile salts, and osmotic stress in gastrointestinal environments.

Furthermore, adhesion to the intestinal mucosa epithelium is a prerequisite for the demonstration of a probiotic effect, and bacterial surface proteins are known to be involved in the colonization of the intestinal mucosa [71,73]. Successful intestinal colonization, a distinguishing feature of effective probiotics, is facilitated by adhesion-related genes in the LP28 genome. The *srtA* gene, encoding LPXTG-specific sortase, is absent in ATCC 14917^T^ and WCFS1 but present in the probiotic strain 299v. This sortase recognizes C-terminal LPXTG sorting signals for anchoring surface proteins to peptidoglycan, enabling stable attachment to intestinal epithelial cells and mucus [74]. The LP28 genome contains the *fbaA*, *scpA*, and *scpB* genes, encoding fibronectin-binding proteins that facilitate continuous bacteria–host cell interactions through surface fibronectin binding. These results demonstrated that LP28 possesses a comprehensive set of adhesion-related genes, including *eno*, *fbaA*, *sasA*, *scpA*, *scpB*, and *srtA*, indicating similarity with probiotic strains such as 299v and WCFS1. Consequently, the expression of these genes is likely to facilitate the colonization of LP28 on the intestinal epithelial cells of the host. In addition, the fact that LP28 possesses the same gene sets as the well-known probiotic strains 299v and WCFS1 for the above probiotic-related genes strongly suggests that LP28 is likely to exert beneficial effects as a probiotic in humans.

In the genotoxicity assessments, including the Ames test, chromosomal aberration assay, and micronucleus assay, no mutagenic or clastogenic effects were observed, with no significant differences from the negative controls (*p* > 0.05). A 28-day subacute oral toxicity study in rats at doses up to 2000 mg/kg revealed no mortality, behavioral abnormalities, or treatment-related histopathological lesions; minor, non-dose-dependent variations in organ weights and blood parameters remained within physiological ranges. Moreover, LP28 demonstrated nonhemolytic (*γ*-hemolytic) activity. LP28 exhibited resistance to kanamycin, which is consistent with the findings of previous studies [75,76,77]. Many *Lactobacillus* species are known to exhibit resistance to aminoglycoside antibiotics, including gentamicin, kanamycin, and streptomycin, demonstrating that nonsynonymous mutations in genes encoding major facilitator family proteins, ABC transporter substrate-binding proteins, and histidine kinases significantly increase kanamycin resistance in *L. plantarum* [78,79]. A comprehensive genome-wide search for AMR genes, along with an analysis of potential gene transfer, mobile elements, VFs, and toxin-related genes within the LP28 genome, revealed no evidence of the presence of any AMR genes or horizontal transfer risks. The absence of virulence factors, toxin genes, and biogenic amine production pathways further validated the safety of LP28.

In this study, we analyzed the whole-genome sequence of LP28, which is highly homologous (ANI ≥ 98.76%) to those of the reference strains ATCC 14917^T^, 299v, and WCFS1. At the genomic level, LP28 demonstrates versatile metabolic functions for environmental adaptation, with its CAZyme profile indicating robust carbohydrate utilization capabilities. We verified key probiotic characteristics, including stress resistance, adhesion ability, bacteriocin biosynthesis, and the absence of virulence or antibiotic resistance genes, through comprehensive in silico safety analysis. Safety for humans and animals was further assessed via phenotypic and oral toxicity analyses. While our previous in vivo study confirmed its efficacy in animal models, the lack of comprehensive in vitro phenotypic evaluations to confirm these genomic predictions remains a limitation. Future in vitro assessments and human clinical trials are required to fully characterize the probiotic properties of *L. plantarum* LP28. These findings establish LP28 as a safe and promising probiotic candidate suitable for various applications in the probiotic industry, including fermented food production and functional health products.

## 5. Conclusions

In this study, a combination approach integrating genomic analysis, in vitro tests, and in vivo experiments was used to assess the safety and potential probiotic properties of LP28 as a probiotic for human consumption. These included whole-genome sequence analysis, an assessment of hemolytic activity, an antimicrobial susceptibility test, the Ames bacterial reverse mutation assay, a chromosomal aberration test in CHO-K1 cells, and a peripheral blood micronucleus test in ICR mice. The results demonstrated that LP28 is both nonhemolytic and susceptible to all antibiotics (except kanamycin) prescribed for *L. plantarum* by the EFSA and has no mutagenic or genotoxic potential. A 28-day repeated-dose oral toxicity test in SD rats confirmed that LP28 has no subacute toxicity at a dosage of 2000 mg/kg body weight/day. Our findings based on a comparative genomic analysis revealed that the genome of LP28 is highly similar to those of *L. plantarum* ATCC 14917^T^, 299v, and WCFS1. In addition, LP28 contains a comprehensive set of stress response genes that facilitate resistance to acid, bile salt, heat, osmotic, and oxidative stresses, as well as adhesion-related genes. Notably, LP28 harbors six genes (*plnA*, *plnE*, *plnF*, *plnJ*, *plnK*, and *plnN*) that encode bacteriocin precursor peptides, suggesting the potential for enhanced probiotic effects through the production of antimicrobial plantaricins. Furthermore, the LP28 genome lacks antimicrobial resistance genes (including resistance against kanamycin), virulence factors, toxin-related genes, biogenic amine synthesis genes, and transferable antibiotic resistance genes. These findings are consistent with the safety profiles of WCFS1 and 299v, both of which are recognized as safe probiotic strains. Overall, LP28 is considered safe and possesses the potential to exert beneficial probiotic properties. Further studies could be conducted to elucidate the application potential of LP28 for both gastrointestinal health in humans and animals and in the food industry.

## Figures and Tables

**Table 1:** Genes encoding proteins involved in stress resistance.

Stress Response	Product	LP28	ATCC14917^T^	299v	WCFS1
Bile resistance	Choloylglycine hydrolase	LP28_02863	ATCC14917_02615	299v_00842	WCFS1_02882
Acid response	ATP synthase epsilon chain	LP28_01986	ATCC14917_01236	299v_02655	WCFS1_02050
	ATP synthase subunit beta	LP28_01987	ATCC14917_01235	299v_02654	WCFS1_02051
	ATP synthase gamma chain	LP28_01988	ATCC14917_01234	299v_02653	WCFS1_02052
	ATP synthase subunit alpha	LP28_01989	ATCC14917_01233	299v_02652	WCFS1_02053
	ATP synthase subunit delta	LP28_01990	ATCC14917_01232	299v_02651	WCFS1_02054
	ATP synthase subunit b	LP28_01991	ATCC14917_01231	299v_02650	WCFS1_02055
	ATP synthase subunit c	LP28_01992	ATCC14917_01230	299v_02649	WCFS1_02056
	ATP synthase subunit a	LP28_01993	ATCC14917_01229	299v_02648	WCFS1_02057
Heat stress	Chaperone protein ClpB	LP28_01581	ATCC14917_00383	299v_02425	WCFS1_01642
	Chaperone protein DnaJ	LP28_01668	ATCC14917_00478	299v_00514	WCFS1_01744
	Chaperone protein DnaK	LP28_01669	ATCC14917_00479	299v_00513	WCFS1_01745
	33 kDa chaperonin	LP28_00458	ATCC14917_01854	299v_01874	WCFS1_00467
	Co-chaperonin GroES	LP28_00562	ATCC14917_00777	299v_01114	WCFS1_00645
	Chaperonin GroEL	LP28_00563	ATCC14917_00778	299v_01113	WCFS1_00646
Osmotic pressure	Carnitine transport ATP-binding protein OpuCA	LP28_01329	ATCC14917_00132	299v_00178	WCFS1_01395
	Carnitine transport permease protein OpuCB	LP28_01330	ATCC14917_00133	299v_00177	WCFS1_01396
	Glycine betaine/carnitine/choline-binding protein OpuCC	LP28_01331	ATCC14917_00134	299v_00176	WCFS1_01397
	Carnitine transport permease protein OpuCD	LP28_01332	ATCC14917_00135	299v_00175	WCFS1_01398
Oxidative stress	Glutaredoxin-like protein	LP28_00535	ATCC14917_00744	299v_01141	WCFS1_00618
	Thioredoxin reductase	LP28_00597	ATCC14917_00811	299v_01080	WCFS1_00673
	Thiol peroxidase	LP28_01947	ATCC14917_01275	299v_02543	WCFS1_02018
Cell adherence	Enolase	LP28_00624/LP28_01596	ATCC14917_00002/ ATCC14917_00398/ ATCC14917_00838	299v_02410/ 299v_01053	WCFS1_00700/ WCFS1_01657
	Fructose-bisphosphate aldolase	LP28_00283	ATCC14917_01683	299v_00801	WCFS1_00281
	SasA, LPXTG motif	LP28_02089/LP28_01362/LP28_02609/LP28_02562	n.d.	299v_00144/299v_01413/299v_01845/299v_02726	n.d.
	Segregation and condensation protein A	LP28_01572	ATCC14917_00373	299v_02434	WCFS1_01633
	Segregation and condensation protein B	LP28_01571	ATCC14917_00372	299v_02435	WCFS1_01632
	Sortase A, LPXTG specific	LP28_00422	n.d.	299v_01910	n.d.

n.d., not detected.

**Table 2:** Mutagenicity of LP28 as assessed by the Ames test using *Salmonella Typhimurium* strains.

Number of Revertant Colonies/Plate															
*S.* Typhimurium Strain															
	TA98			TA100			TA102			TA1535			TA1537		
**With S9mix**															
Negative control ^a^	43	±	5	226	±	12	404	±	7	15	±	2	13	±	3
Positive control ^b^	251	±	3 ****	856	±	5 ****	994	±	5 ****	187	±	3 ****	290	±	7 ****
LP28 (5.0000 mg)	44	±	3	206	±	8	417	±	8	13	±	3	14	±	2
LP28 (2.5000 mg)	45	±	3	210	±	2	427	±	8 **	14	±	5	11	±	2
LP28 (1.2500 mg)	43	±	3	220	±	9	429	±	3 **	12	±	3	11	±	2
LP28 (0.6250 mg)	42	±	3	236	±	9	416	±	8	16	±	2	11	±	1
LP28 (0.3125 mg)	44	±	2	245	±	4 *	437	±	8 ***	15	±	3	12	±	1
**Without S9mix**															
Negative control ^a^	43	±	3	219	±	8	378	±	2	19	±	9	10	±	2
Positive control ^c^	254	±	9 ****	659	±	6 ****	911	±	8 ****	188	±	6 ****	289	±	9 ****
LP28 (5.0000 mg)	38	±	6	183	±	8	365	±	5	18	±	3	12	±	4
LP28 (2.5000 mg)	43	±	4	206	±	9	380	±	5	13	±	3	11	±	2
LP28 (1.2500 mg)	44	±	3	198	±	3	374	±	9	15	±	2	11	±	2
LP28 (0.6250 mg)	45	±	7	197	±	5	332	±	12	17	±	3	10	±	1
LP28 (0.3125 mg)	44	±	3	237	±	10 *	390	±	8	16	±	3	11	±	2

The data are shown as the mean ± SD. One-way ANOVA with Dunnett’s post hoc test was performed to compare each group with the negative control group. Asterisks indicate significant differences (* *p* < 0.05, ** *p* < 0.01, *** *p* < 0.001, **** *p* < 0.0001). ^a^ Sterile water was used as a negative control. ^b^ Positive controls with S9 for TA98: benzo[α]pyrene, 4 μg/plate; for TA100: 2-aminoanthracene, 4 μg/plate; for TA102: 2-aminoanthracene, 10 μg/plate; for TA1535: 2-aminoanthracene, 4 μg/plate; for TA1537: 2-aminoanthracene, 4 μg/plate. ^c^ Positive controls without S9 for TA98: 4-nitroquinoline 1-oxide, 0.5 μg/plate; for TA100: sodium azide, 5.0 μg/plate; for TA102: mitomycin C, 0.5 μg/plate; for TA1535: sodium azide, 0.4 μg/plate; for TA1537: 9-aminoanthracene, 50.0 μg/plate.

**Table 3:** Chromosomal aberration test for LP28 in CHO-K1 cells.

	Number of Chromosome Aberrations									Total Number of Chromosomal Aberrations (%)	Number of Cells with Chromosomal Aberrations (%) ^d^
	(Per 100 Cells)										
	G	B	D	R	g	b	Int	Itr	Other		
**3 h with S9 mix**											
Negative control ^a^	0.3	0.7	0.0	0.0	2.3	0.7	0.0	0.0	0.0	4.0	0.7
Positive control ^b^	3.7	3.7	0.7	1.7	6.7	5.3	0.0	0.0	0.0	21.7 ****	13.3 ****
LP28 (2.0 mg/mL)	0.3	0.3	0.0	0.0	2.0	2.0	0.0	0.0	0.0	4.7	2.3
LP28 (1.0 mg/mL)	1.0	0.7	0.0	0.0	3.0	1.7	0.0	0.0	0.0	6.3	1.3
LP28 (0.5 mg/mL)	0.7	0.3	0.0	0.0	3.3	1.7	0.0	0.0	0.0	6.0	1.3
**3 h without S9 mix**											
Negative control ^a^	1.7	0.3	0.0	0.3	2.7	2.3	0.0	0.0	0.0	7.3	1.3
Positive control ^c^	3.7	3.0	1.0	2.3	5.7	4.3	0.0	0.0	0.0	20.0 ****	13.3 ****
LP28 (2.0 mg/mL)	1.3	0.7	0.0	0.0	2.7	1.7	0.0	0.0	0.0	6.3	1.7
LP28 (1.0 mg/mL)	0.0	0.3	0.0	0.0	3.3	1.0	0.0	0.0	0.0	4.7	1.3
LP28 (0.5 mg/mL)	0.3	0.7	0.0	0.0	2.7	2.0	0.0	0.0	0.0	5.7	2.0
**20 h without S9 mix**											
Negative control ^a^	0.3	0.3	0.0	0.0	3.3	2.0	0.0	0.0	0.0	6.0	2.3
Positive control ^c^	2.3	2.3	1.0	1.7	7.3	5.0	0.0	0.0	0.0	19.7 ****	12.7 ****
LP28 (2.0 mg/mL)	0.3	0.0	0.0	0.0	3.7	2.7	0.0	0.0	0.0	6.7	1.7
LP28 (1.0 mg/mL)	0.3	0.3	0.0	0.0	3.3	1.3	0.0	0.0	0.0	5.3	1.3
LP28 (0.5 mg/mL)	0.3	0.3	0.0	0.0	4.7	2.0	0.0	0.0	0.0	7.3	2.3

The data are shown as the mean of three independent replicates. Statistical significance between each treatment group and the negative control was determined using Fisher’s exact test. Asterisks indicate significant differences compared with the negative control (**** *p* < 0.0001). ^a^ Ham’s F-12 medium with 10% fetal bovine serum. ^b^ 25 μg/mL cyclophosphamide monohydrate was used with S9. ^c^ 2.5 μg/mL mitomycin C was used without S9. ^d^ G, chromosome gap; B, chromosome break; D, dicentric; R, ring; g, chromatid gap; b, chromatid break; Int, interchange; Itr, intrachange; other, polyploid or centromeric disruption.

**Table 4:** Assessment of the reticulocyte ratio and micronucleus incidence in the peripheral blood of LP28-treated ICR mice.

	Reticulocyte Ratio ^1^			Micronucleus Incidence ^2^		
	RETs/2000 RBCs (‰)			Mn-RETs/4000 RETs (‰)		
	Mean ± SD			Mean ± SD		
Negative control ^3^	45.2	±	1.8 ^a^	0.1	±	0.1 ^a^
Positive control ^4^	17.6	±	2.8 ^b^	16.3	±	3.2 ^b^
LP28 (500 mg/kg BW)	43.9	±	3.3 ^ab^	0.1	±	0.1 ^a^
LP28 (1000 mg/kg BW)	45.8	±	4.1 ^a^	0.1	±	0.1 ^ab^
LP28 (2000 mg/kg BW)	43.4	±	1.9 ^ab^	0.0	±	0.0 ^a^

The data are shown as the mean ± SD. Statistical differences among groups were evaluated using the Kruskal–Wallis test with Dunn’s post hoc test. Different letters (^a^, ^b^) indicate a statistically significant difference between groups (*p* < 0.05). ^1^ Reticulocyte ratio: reticulocytes (RETs) per 2000 red blood cells (RBCs); *n* = 5. ^2^ Micronucleus incidence: micronucleated reticulocytes (Mn-RETs) per 4000 reticulocytes; *n* = 5. ^3^ Sterile water was used as the negative control. ^4^ Cyclophosphamide monohydrate (50 mg/kg body weight) was used as the positive control.

**Table 5:** Body and organ weights of Sprague–Dawley rats following 28 days of LP28 administration.

	Male											
	Control			Low Dose (500 mg LP28/kg BW)			Medium Dose (1000 mg LP28/kg BW)			High Dose 2000 mg LP28/kg BW)		
Body weight (before)	201.3	±	8.7 ^a^	199.3	±	8.3 ^a^	200.3	±	11.0 ^a^	202.0	±	10.6 ^a^
Body weight (after)	386.2	±	14.3 ^a^	366.6	±	11.7 ^a^	374.7	±	8.3 ^a^	377.0	±	29.4 ^a^
Testis	3.128	±	0.261 ^a^	3.259	±	0.141 ^a^	3.183	±	0.219 ^a^	3.326	±	0.240 ^a^
Adrenal gland	0.050	±	0.010 ^a^	0.066	±	0.009 ^b^	0.059	±	0.006 ^ab^	0.068	±	0.010 ^b^
Spleen	0.698	±	0.133 ^a^	0.674	±	0.069 ^a^	0.677	±	0.071 ^a^	0.712	±	0.096 ^a^
Kidney	3.388	±	0.289 ^a^	3.183	±	0.144 ^a^	3.498	±	0.462 ^a^	3.440	±	0.264 ^a^
Heart	1.482	±	0.125 ^a^	1.408	±	0.092 ^a^	1.529	±	0.109 ^a^	1.387	±	0.123 ^a^
Brain	2.045	±	0.110 ^a^	1.944	±	0.058 ^a^	2.056	±	0.032 ^a^	2.011	±	0.134 ^a^
Liver	15.301	±	1.774 ^a^	13.804	±	1.138 ^a^	14.797	±	1.693 ^a^	13.868	±	1.104 ^a^
	**Female**											
	**Control**			**Low Dose** **(500 mg LP28/kg BW)**			**Medium Dose** **(1000 mg LP28/kg BW)**			**High Dose** **(2000 mg LP28/kg BW)**		
Body weight (before)	176.8	±	10.8 ^a^	176.4	±	10.4 ^a^	175.0	±	9.6 ^a^	174.9	±	10.3 ^a^
Body weight (after)	223.4	±	20.9 ^a^	218.8	±	16.1 ^a^	224.4	±	19.2 ^a^	216.5	±	13.9 ^a^
Ovary	0.102	±	0.026 ^a^	0.109	±	0.024 ^a^	0.110	±	0.031 ^a^	0.105	±	0.015 ^a^
Adrenal gland	0.070	±	0.013 ^a^	0.065	±	0.004 ^a^	0.073	±	0.007 ^a^	0.064	±	0.010 ^a^
Spleen	0.476	±	0.067 ^a^	0.461	±	0.041 ^a^	0.500	±	0.044 ^a^	0.487	±	0.054 ^a^
Kidney	1.954	±	0.240 ^a^	1.828	±	0.370 ^a^	1.984	±	0.164 ^a^	1.999	±	0.164 ^a^
Heart	0.894	±	0.041 ^a^	0.904	±	0.062 ^a^	0.908	±	0.109 ^a^	0.883	±	0.071 ^a^
Brain	1.837	±	0.095 ^a^	1.823	±	0.087 ^a^	1.820	±	0.091 ^a^	1.867	±	0.114 ^a^
Liver	8.229	±	1.312 ^a^	7.581	±	0.795 ^a^	8.654	±	0.882 ^a^	7.267	±	0.520 ^a^

The data are shown as the mean ± SD; *n* = 12. Statistical differences in body weight and absolute organ weights among groups were analyzed using one-way ANOVA with Tukey’s post hoc test. Different letters (^a^, ^b^) indicate statistically significant differences between groups (*p* < 0.05), whereas identical letters indicate no statistically significant difference between groups (*p* > 0.05). BW, body weight.

**Table 6:** Antimicrobial susceptibility of LP28.

Antibiotic	MIC ^a^	Cut-Off Value ^b^	Sensitivity ^c^	Antimicrobial Resistance Gene
Ampicillin	<0.5	2	S	ND ^d^
Gentamicin	8	16	S	ND
Kanamycin	128	64	R	ND
Erythromycin	<0.5	1	S	ND
Clindamycin	<1	4	S	ND
Tetracycline	<16	32	S	ND
Chloramphenicol	1	8	S	ND

^a^ MIC, minimal inhibitory concentration (mg/L) toward LP28. ^b^ Microbiological cutoff values (mg/L) for antibiotics recommended by the European Food Safety Authority. ^c^ S, Susceptible; R, Resistant. ^d^ ND, Not detected.

## Data Availability

The raw data supporting the conclusions of this article will be made available by the authors on request.

## Supplementary Materials

The following supporting information can be downloaded at: https://www.mdpi.com/article/10.3390/microorganisms14040843/s1. Figure S1: Phylogenetic tree based on 16S rRNA gene sequences showing the relationship between strain LP28 and type strains of closely related species within the genus *Lactiplantibacillus*; Figure S2: Phylogenetic tree based on *pheS* gene sequences showing the relationship between strain LP28 and type strains of closely related species within the genus *Lactiplantibacillus*; Figure S3: Phylogenomic tree based on TYGS results showing the relationship between the strain LP28 and its closely related type strains in the genus; Figure S4: Comparative genomics of *Lactiplantibacillus plantarum* strains showing CAZy family distributions; Figure S5: Hemolytic activity of LP28; Table S1: Sequence similarity of LP28 and its closely related type strains; Table S2: Average nucleotide identity (ANI) values and digital DNA–DNA hybridization (dDDH) prediction values between strain LP28 and the type strains of closely related species in the genus *Lactiplantibacillus*; Table S3: Comparison of the genomic characteristics of *L. plantarum* strains LP28, ATCC 14917^T^, 299v, and WCFS1; Table S4. Comparison of SEED subsystem features of LP28 and *L. plantarum* reference strains, ATCC 14917^T^, 299v, and WCFS1. Functional roles of RAST-annotated genes were assigned by the RAST annotation server. The subsystem features for each genome were obtained using the SEED Viewer server; Table S5: Cluster of Orthologous Groups (COG) functional categories of identified protein-coding genes in the genomes of LP28, ATCC 14917^T^, 299v, and WCFS1; Table S6: Specific functional clusters of LP28; Table S7: Hematological profiles of male and female Sprague–Dawley rats following 28-day oral administration of LP28; Table S8: Serum biochemical parameters in male and female Sprague-Dawley rats following 28-day oral administration of LP28; Table S9: Antimicrobial Resistance genes analysis of LP28; Table S10. List of insertion sequences (IS) identified in the genome of LP28; Table S11: Bioinformatic analysis of phage sequences in the genome of LP28.
