# Supplementary material for: Assessment of the Safety and Potential Probiotic Properties of Lactiplantibacillus plantarum LP28 Based on Whole Genome Sequencing and Phenotypic and Oral Toxicity Analyses
Source: Microorganisms. 2026 Apr 9;14(4):843. doi: 10.3390/microorganisms14040843 (PMC13119284; doi:10.3390/microorganisms14040843)
Supplement: Supplementary file 1 [file microorganisms-14-00843-s001.zip › microorganisms-4198639-supplementary.pdf]

Microorganisms Supplementary materials for:

Assessment of the Safety and Potential Probiotic Properties of *Lactiplantibacillus plantarum* LP28 Based on Whole Genome Sequencing and Phenotypic and Oral Toxicity Analyses

Yi-Chu Liao[1*], Yi-Chen Cheng[1], Chia-Chia Lee[1], Yun-Fang Cheng[1], Han-Yin Hsu[1], Shih-Hsuan Lin[1], Jin-Seng Lin[1], San-Land Young[1], Koichi Watanabe[1,2,3*]

[1] Culture Collection & Research Institute, SYNBIO TECH INC., Kaohsiung, Taiwan;
[2] Bioresource Collection and Research Center, Food Industry Research and Development Institute, Hsin-chu, Taiwan
[3] Department of Animal Science and Technology, National Taiwan University, Taipei, Taiwan

*Correspondence:
Yi-Chu Liao (yc.liao@synbiotech.com.tw)
Koichi Watanabe (koichi_wtnb@yahoo.co.jp)

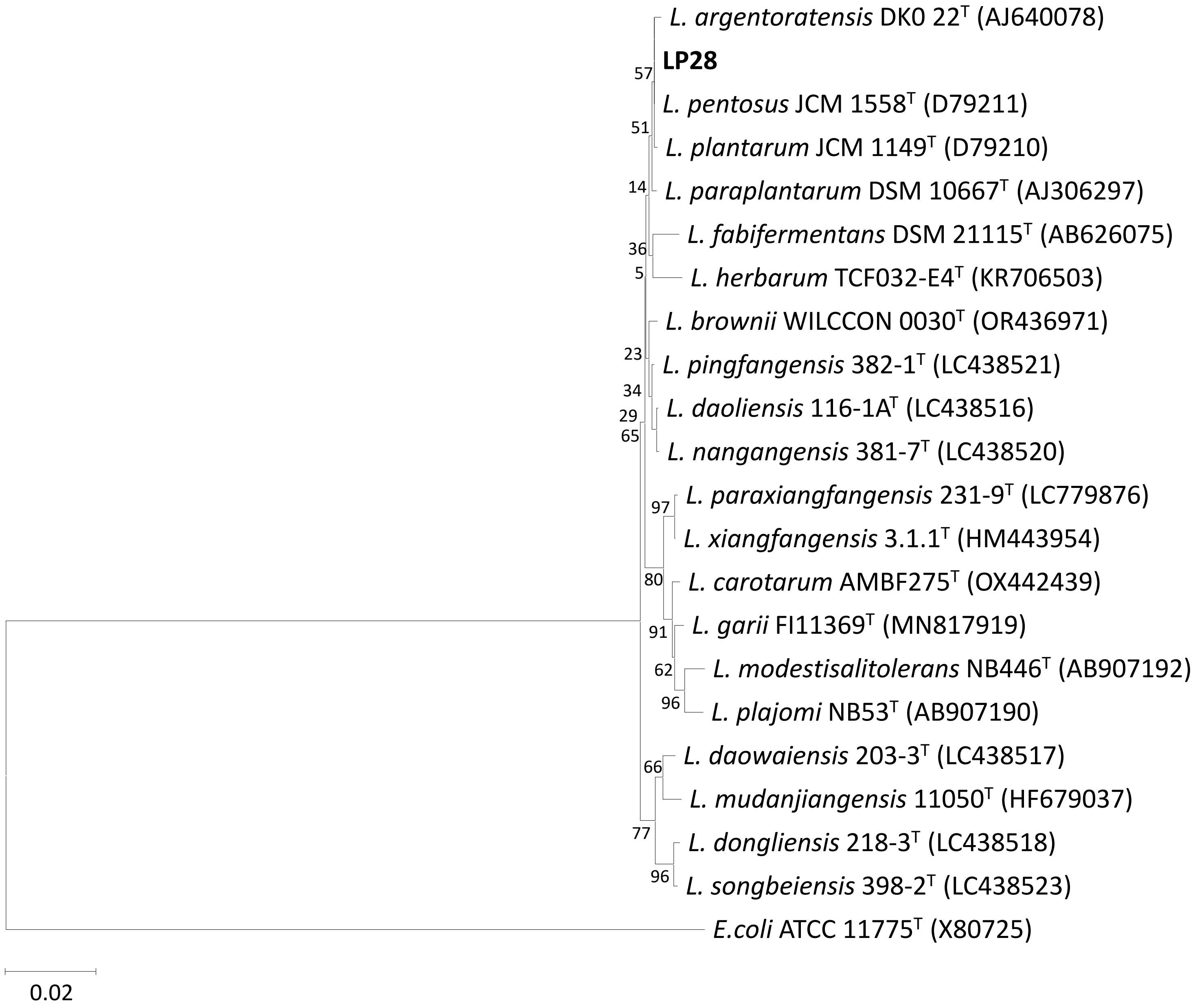

Figure S1. Phylogenetic tree based on 16S rRNA gene sequences showing the relationship between strain LP28 and type strains of closely related species within the genus *Lactiplantibacillus*. The tree was constructed using the neighbor-joining method. *Escherichia coli* ATCC 11775^T was used as an outgroup. Bootstrap values based on 1,000 replications are indicated at the nodes. GenBank accession numbers are shown in parentheses. Bar, 2% sequence divergence. Phylogenetic tree was constructed with MEGA12 software.

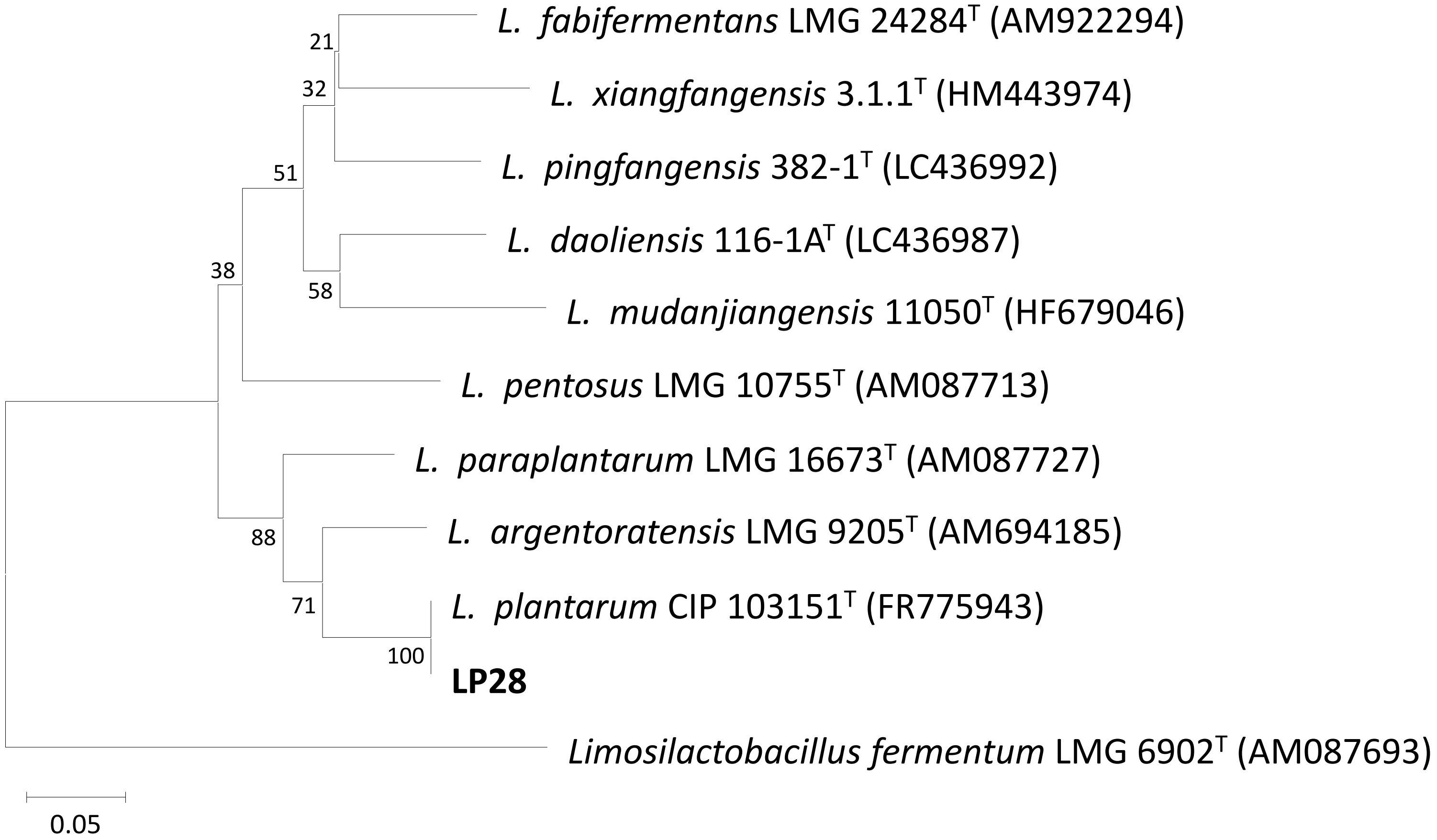

Figure S2. Phylogenetic tree based on *pheS* gene sequences showing the relationship between strain LP28 and type strains of closely related species within the genus *Lactiplantibacillus*. The tree was constructed using the neighbor-joining method. *Limosilactobacillus fermentum* LMG 6902^T was used as an outgroup. Bootstrap values based on 1,000 replications are indicated at the nodes. GenBank accession numbers are shown in parentheses. Bar, 5% sequence divergence. Phylogenetic tree was constructed with MEGA 12 software.

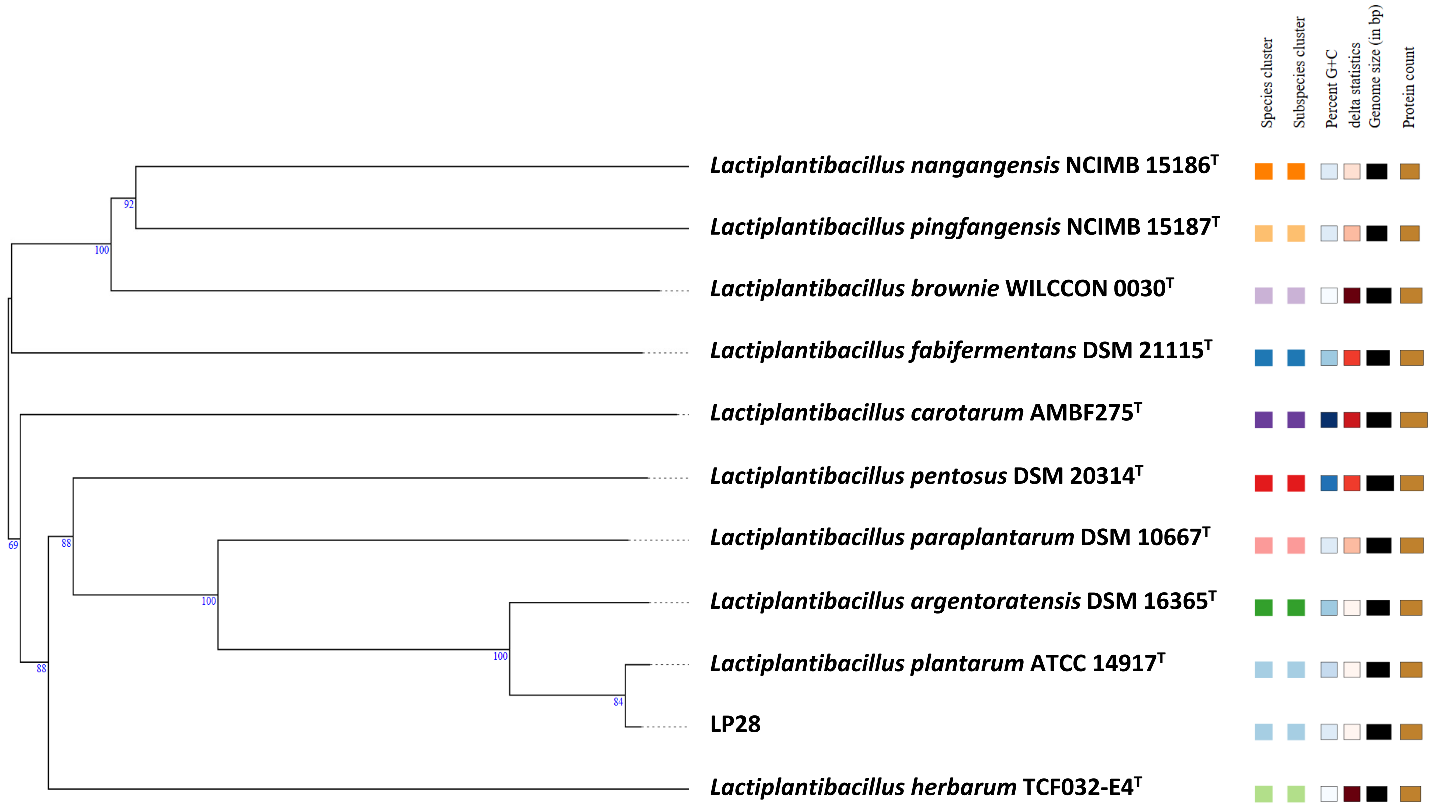

Figure S3. Phylogenomic tree based on TYGS results showing the relationship between the strain LP28 and its closely related type strains in the genus. The tree was inferred with FastME 2.1.6.1 from GBDP distances calculated from genome sequences. The branch lengths are scaled in terms of GBDP distance. The numbers above branches represent GBDP pseudo-bootstrap support values greater than 60% from 100 replications.

CAZy family		Strain			
	Subcategory	LP28	ATCC 14917^T	299v	WCFS1
Auxiliary Activities	AA10	3	3	3	3
Carohydrate-Binding Modules	CBM50	16	14	14	14
Cabohydrate Esterases	CE9	1	1	1	1
	CE12	1	1	1	1
Glycoside Hydrolases	GH1	22	22	24	22
	GH3	1	1	1	1
	GH5	1	1	1	1
	GH8	1	0	0	0
	GH12	2	2	2	1
	GH13	26	26	25	25
	GH20	1	1	1	1
	GH23	7	7	6	7
	GH24	1	1	1	1
	GH25	9	8	8	10
	GH27	1	1	1	1
	GH28	3	3	3	3
	GH31	1	1	1	1
	GH32	2	2	2	2
	GH36	5	2	3	2
	GH38	3	3	3	2
	GH39	0	0	0	1
	GH42	3	3	3	3
	GH65	10	10	11	10
	GH73	4	6	4	6
	GH78	3	4	4	3
	GH84	1	1	1	1
	GH85	0	1	0	0
	GH92	3	3	3	3
	GH125	1	1	1	1
	GH126	1	1	1	0
	GH170	3	4	3	5
	Subtotal in GH	115	115	113	113
Glycosyl Transferases	GT0	1	1	1	1
	GT1	0	0	0	1
	GT2	27	29	27	25
	GT4	25	28	28	31
	GT5	2	2	2	2
	GT26	2	2	2	2
	GT28	2	2	2	2
	GT30	1	1	1	1
	GT32	0	1	0	1
	GT36	1	1	1	1
	GT47	1	1	1	1
	GT51	4	4	4	4
	GT58	1	1	1	1
	GT83	1	1	1	1
	GT111	2	1	2	2
	GT113	0	1	0	0
	Subtotal in GT	70	76	73	76
	Total	206	210	205	208

Count
- >30
- 20–30
- 10–20
- 0–10
- 0

Figure S4. Comparative genomics of *Lactiplantibacillus plantarum* strains showing CAZy family distributions. A heatmap illustrates dbCAN2 annotations of Auxiliary Activities (AAs), Carbohydrate-Binding Modules (CBMs), Carbohydrate Esterases (CEs), Glycoside Hydrolases (GHs), and Glycosyl Transferases (GTs) families from the CAZy database. Yellow indicates the absence of a family, while a light-blue to dark-blue gradient represents an increasing number of members in that family.

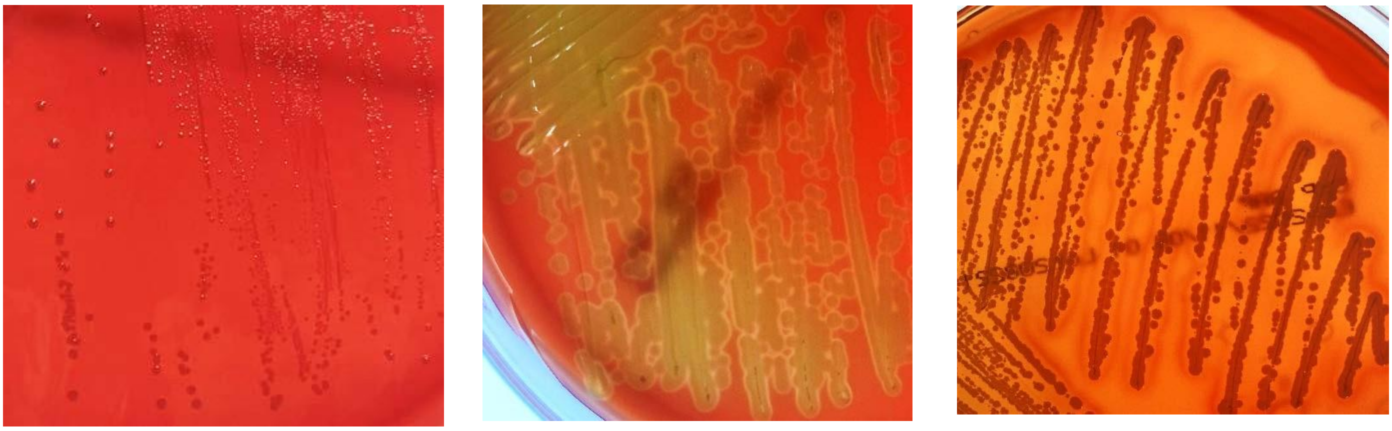

(a) **(b)** **(c)**

Figure S5. Hemolytic activity of LP28. Hemolytic activity was assessed by culturing the strains on blood agar supplemented with 5% (w/v) defibrinated sheep blood. Plates were incubated at 37 °C under anaerobic conditions for 48 h. Representative hemolysis patterns are shown as follows: (A) LP28; (B) α-hemolytic positive control, *Streptococcus pneumoniae* ATCC 6305; and (C) β-hemolytic positive control, *Staphylococcus aureus* ATCC 25923.

Table S1. Sequence similarity of LP28 and its closely related type strains.

Strain	Sequence similarity (%) with LP28	
	16S rRNA	*pheS*
Lactiplantibacillus plantarum JCM 1149[T]	99.9	100
Lactiplantibacillus pentosus JCM 1558[T]	100	79.0
Lactiplantibacillus argentoratensis DK0 22[T]	99.8	89.6
Lactiplantibacillus paraplantarum DSM 10667[T]	99.8	89.2
Lactiplantibacillus pingfangensis 382-1[T]	99.6	77.6
Lactiplantibacillus daoliensis 116-1A[T]	99.5	75.0
Lactiplantibacillus fabifermentans DSM 21115[T]	99.2	76.1
Lactiplantibacillus xiangfangensis 3.1.1[T]	99.0	73.5
Lactiplantibacillus mudanjiangensis 11050[T]	98.8	75.6

Table S2. Average nucleotide identity (ANI) values and digital DNA–DNA hybridization (dDDH) prediction values between strain LP28 and the type strains of closely related species in the genus *Lactiplantibacillus*.

	Species	Strain	Accession No.	1	2	3	4	5	6	7	8	9	10	11
1	*Lactiplantibacillus plantarum*	LP28		*	94.1	62.6	31.6	24.1	22.5	22.1	22.7	23.4	20.9	20.6
2	*Lactiplantibacillus plantarum*	ATCC 14917[T]	GCA_000143745.1	99.07	*	62.8	31.1	23.8	22.4	20.8	21.7	21.5	20.6	20.4
3	*Lactiplantibacillus argentoratensis*	DSM 16365[T]	GCA_001435215.1	94.82	94.93	*	31.2	24.7	21.8	21.9	21.8	23.6	20.9	20.5
4	*Lactiplantibacillus paraplantarum*	DSM 10667[T]	GCA_003641145.1	85.43	85.24	85.15	*	24.4	23.0	22.5	22.1	24.4	21.1	21.2
5	*Lactiplantibacillus pentosus*	DSM 20314[T]	GCA_003641185.1	79.22	79.30	79.52	79.40	*	22.1	22.3	22.0	22.2	21.2	20.8
6	*Lactiplantibacillus herbarum*	TCF032-E4[T]	GCA_001039045.1	76.80	76.8	76.61	77.19	76.49	*	21.2	21.3	20.7	20.3	20.4
7	*Lactiplantibacillus carotarum*	AMBF275[T]	GCA_947539765.1	75.74	75.01	75.91	75.78	75.71	75.23	*	21.9	22.2	20.9	20.7
8	*Lactiplantibacillus fabifermentans*	DSM 21115[T]	GCA_000498955.2	75.20	74.84	75.08	75.27	75.19	74.43	75.39	*	22.3	21.4	21.3
9	*Lactiplantibacillus brownii*	WILCCON 0030[T]	GCA_031085375.1	75.32	74.49	75.59	75.76	75.05	74.43	75.09	76.42	*	24.6	24.1
10	*Lactiplantibacillus nangangensis*	NCIMB 15186[T]	GCA_005405065.1	74.12	74.15	74.24	74.32	74.25	74.21	74.43	75.99	80.35	*	24.4
11	*Lactiplantibacillus pingfangensis*	NCIMB 15187[T]	GCA_005404945.1	74.07	74.05	74.04	74.20	74.2	74.10	74.14	75.65	79.68	80.08	*

The values on the lower left are the OrthoANI values (%), and the values on the upper right are the dDDH values (%).

Table S3. Comparison of the genomic characteristics of *L. plantarum* strains LP28, ATCC 14917^{T}, 299v, and WCFS1.

	Strain			
	**LP28**	**ATCC 14917^{T}**	**299v**	**WCFS1**
Accession No.		GCA_000143745.1	GCA_001888735.1	GCA_000203855.3
Genome size (bp)	3,364,908	3,212,261	3,302,055	3,348,624
G + C content (%)	44.3	44.5	44.4	44.4
No. of contigs	3	9	67	4
N50 length (bp)	3,244,423	152,365	173,004	3,308,273
L50	1	6	8	1
Genes	3,229	3,061	3,164	3,211
CDS	3,144	2,999	3,105	3,123
tRNA	68	59	56	71
rRNA	16	2	3	16
tmRNA	1	1	0	1

bp, base pairs; CDS, coding sequence; tRNA, transfer RNA; rRNA, ribosomal RNA; tmRNA, transfer-messenger RNA.

Table S4. Comparison of SEED subsystem features of LP28 and *L. plantarum* reference strains, ATCC 14917^{T}, 299v, and WCFS1. Functional roles of RAST-annotated genes were assigned by the RAST annotation server. The subsystem features for each genome were obtained using the SEED Viewer server.

	**LP28**		**ATCC 14917^{T}**		**299v**		**WCFS1**	
**Subsystem Feature**	**Counts**	**%**	**Counts**	**%**	**Counts**	**%**	**Counts**	**%**
Carbohydrates	147	17.9	144	17.6	130	17.5	106	13.1
Amino Acids and Derivatives	122	14.9	120	14.7	118	15.9	118	14.5
Protein Metabolism	105	12.8	102	12.5	103	13.8	106	13.1
Cofactors, Vitamins, Prosthetic Groups, Pigments	79	9.6	80	9.8	70	9.4	83	10.2
Nucleosides and Nucleotides	75	9.1	74	9	74	9.9	75	9.2
DNA Metabolism	49	6	47	5.7	50	6.7	54	6.7
RNA Metabolism	35	4.3	36	4.4	32	4.3	34	4.2
Membrane Transport	28	3.4	28	3.4	19	2.6	28	3.4
Virulence, Disease and Defense	28	3.4	28	3.4	26	3.5	28	3.4
Fatty Acids, Lipids, and Isoprenoids	26	3.2	25	3.1	16	2.2	26	3.2
Cell Wall and Capsule	25	3	38	4.6	24	3.2	55	6.8
Respiration	17	2.1	16	2	14	1.9	16	2
Stress Response	15	1.8	16	2	15	2	15	1.8
Regulation and Cell signaling	13	1.6	13	1.6	10	1.3	16	2
Miscellaneous	11	1.3	11	1.3	11	1.5	11	1.4
Phosphorus Metabolism	10	1.2	5	0.6	4	0.5	5	0.6
Phages, Prophages, Transposable elements, Plasmids	9	1.1	9	1.1	6	0.8	6	0.7
Dormancy and Sporulation	6	0.7	6	0.7	1	0.1	6	0.7
Iron acquisition and metabolism	5	0.6	5	0.6	5	0.7	5	0.6
Cell Division and Cell Cycle	4	0.5	4	0.5	4	0.5	4	0.5
Metabolism of Aromatic Compounds	4	0.5	2	0.2	2	0.3	5	0.6
Potassium metabolism	4	0.5	4	0.5	4	0.5	4	0.5
Secondary Metabolism	4	0.5	4	0.5	4	0.5	4	0.5
Sulfur Metabolism	0	0	2	0.2	2	0.3	2	0.2

Table S5. Cluster of Orthologous Groups (COG) functional categories of identified protein-coding genes in the genomes of LP28, ATCC 14917^T, 299v, and WCFS1.

		LP28		ATCC 14917^T		299v		WCFS1	
**COG Category**	**Description**	**Number of genes**	**%**	**Number of genes**	**%**	**Number of genes**	**%**	**Number of genes**	**%**
**K**	Transcription	311	11	296	10.9	310	11.1	310	10.9
**G**	Carbohydrate transport and metabolism	269	9.5	265	9.7	263	9.4	275	9.6
**E**	Amino acid transport and metabolism	225	8	214	7.9	218	7.8	232	8.1
**L**	Replication, recombination and repair	177	6.3	143	5.2	164	5.9	166	5.8
**J**	Translation, ribosomal structure and biogenesis	172	6.1	170	6.2	169	6.1	171	6
**M**	Cell wall/membrane/envelope biogenesis	163	5.8	171	6.3	178	6.4	173	6.1
**P**	Inorganic ion transport and metabolism	162	5.7	155	5.7	154	5.5	164	5.8
**F**	Nucleotide transport and metabolism	130	4.6	127	4.7	129	4.6	132	4.6
**C**	Energy production and conversion	120	4.3	118	4.3	116	4.2	124	4.4
**H**	Coenzyme transport and metabolism	96	3.4	96	3.5	94	3.4	111	3.9
**U**	Intracellular trafficking, secretion, and vesicular transport	78	2.8	74	2.7	76	2.7	74	2.6
**T**	Signal transduction mechanisms	72	2.6	71	2.6	73	2.6	71	2.5
**I**	Lipid transport and metabolism	66	2.3	64	2.3	66	2.4	68	2.4
**V**	Defense mechanisms	64	2.3	59	2.2	67	2.4	61	2.1
**O**	Posttranslational modification, protein turnover, chaperones	55	1.9	54	2	54	1.9	53	1.9
**D**	Cell cycle control, cell division, chromosome partitioning	38	1.3	37	1.4	43	1.5	40	1.4
**Q**	Secondary metabolite biosynthesis, transport and catabolism	31	1.1	30	1.1	28	1	33	1.2
**N**	Cell motility	15	0.5	16	0.6	13	0.5	15	0.5
**S**	Function unknown	579	20.5	565	20.7	574	20.6	577	20.2
**Total**		2823	100	2725	100	2789	100	2850	100

Table S6. Specific functional clusters of LP28.

ID	Protein Count	Swiss-Prot Hit	GO Annotation
cluster2845	2	N/A	N/A
cluster2846	2	P54585	GO:0022857; F:transmembrane transporter activity; IEA:InterPro
cluster2847	2	N/A	N/A
cluster2848	2	N/A	N/A

N/A: not affiliated

Table S7. Hematological profiles of male and female Sprague–Dawley rats following 28-day oral administration of LP28.

	Male			
	Control	Low Dose (500 mg LP28/kg BW)	Medium Dose (1000 mg LP28/kg BW)	High Dose (2000 mg LP28/kg BW)
WBC (10^3 /μL)	5.5 ± 2.2 [a]	4.9 ± 1.4 [a]	4.4 ± 0.7 [a]	4.3 ± 0.7 [a]
RBC (10^6/μL)	9.1 ± 0.8 [a]	8.4 ± 1.3 [a]	8.7 ± 0.8 [a]	7.9 ± 0.3 [a]
Hemoglobin (g/dL)	17.7 ± 1.7 [a]	17.3 ± 1.5 [a]	16.8 ± 1.1 [a]	15.7 ± 0.6 [a]
Hematocrit (%)	55.7 ± 4.6 [a]	52.3 ± 7.8 [a]	53.4 ± 3.6 [a]	48.8 ± 1.9 [a]
MCV (fL)	62.3 ± 2.4 [a]	62.7 ± 2.0 [a]	61.8 ± 2.8 [a]	62.0 ± 0.8 [a]
MCH (pg)	19.6 ± 0.6 [a]	21 ± 2.7 [a]	19.4 ± 1.1 [a]	20.0 ± 0.5 [a]
MCHC (g/dL)	31.7 ± 0.9 [a]	33.5 ± 4.5 [a]	31.5 ± 0.5[a]	32.2 ± 0.6 [a]
Platelet (10^3/μL)	1118.3 ± 173.4 [a]	1090.8 ± 253.1 [a]	1098.7 ± 194.5 [a]	1353.8 ± 406.2 [a]
Neutrophil (%)	3.1 ± 6.6 [a]	0.6 ± 0.6 [a]	4.3 ± 4.9 [a]	3.5 ± 4.1 [a]
Lymphocyte (%)	94.8 ± 7.8 [a]	95.3 ± 6.0 [a]	93.4 ± 6.9 [a]	94.4 ± 5.6 [a]
Monocyte (%)	0.8 ± 0.6 [a]	3.2 ± 5.9 [a]	0.5 ± 0.3 [a]	0.5 ± 0.4 [a]
Eosinophil (%)	1.1 ± 0.6 [a]	1.0 ± 1.4 [a]	0.6 ± 0.5 [a]	0.8 ± 0.7 [a]
Basophil (%)	0.2 ± 0.5 [a]	0.0 ± 0.1 [a]	1.2 ± 2.3 [a]	0.7 ± 1.2 [a]
PT (sec.)	15.6 ± 2.5 [a]	14.4 ± 2.1 [a]	13.9 ± 2.8 [a]	13.7 ± 1.8 [a]
	Female			
	Control	Low Dose (500 mg LP28/kg BW)	Medium Dose (1000 mg LP28/kg BW)	High Dose (2000 mg LP28/kg BW)
WBC (10^3 /μL)	4.3 ± 2.7 [a]	2.7 ± 0.7[a]	2.9 ± 0.4 [a]	4.7 ± 2.6 [a]
RBC (10^6/μL)	8.4 ± 0.8 [a]	8.5 ± 0.4 [a]	8.5 ± 0.4 [a]	8.5 ± 0.7 [a]
Hemoglobin (g/dL)	16.1 ± 1.2 [a]	16.3 ± 0.9 [a]	16.4 ± 0.8 [a]	16.4 ± 1.1 [a]
Hematocrit (%)	49.9 ± 3.7 [a]	50.3 ± 2.2 [a]	50.5 ± 2.5 [a]	50.0 ± 2.7 [a]
MCV (fL)	59.7 ± 1.6 [a]	58.9 ± 1.0 [a]	59.7 ± 1.3 [a]	59.1 ± 2.1 [a]
MCH (pg)	19.2 ± 0.6 [a]	19 ± 0.5 [a]	19.4 ± 0.3 [a]	19.3 ± 0.6 [a]
MCHC (g/dL)	32.2 ± 0.3 [a]	32.3 ± 0.6 [a]	32.5 ± 0.4 [a]	32.7 ± 0.4 [a]
Platelet (10^3/μL)	1170 ± 259.9 [a]	1013.5 ± 133.2 [a]	1088.2 ± 174.7 [a]	1090.3 ± 223.2 [a]
Neutrophil (%)	1.0 ± 1.6 [a]	2.6 ± 5.8 [a]	1.2 ± 1.4 [a]	1.7 ± 3.0 [a]
Lymphocyte (%)	97.8 ± 1.9 [a]	94.5 ± 7.8 [a]	97.5 ± 1.3 [a]	97.5 ± 3.0 [a]
Monocyte (%)	0.5 ± 0.1 [a]	1.1 ± 1.2 [a]	0.4 ± 0.2 [a]	0.4 ± 0.2 [a]
Eosinophil (%)	0.8 ± 0.5 [ab]	1.5 ± 0.4 [b]	0.9 ± 0.6 [ab]	0.5 ± 0.2 [a]
Basophil (%)	0.0 ± 0.0 [a]	0.3 ± 0.8 [a]	0.0 ± 0.0 [a]	0.0 ± 0.0 [a]
PT (sec.)	10.0 ± 0.6 [a]	9.6 ± 0.7 [a]	9.4 ± 0.5 [a]	10.0 ± 0.3 [a]

Data are shown as mean ± SD, n = 12. Statistical differences among groups were analyzed using one-way ANOVA with Tukey's post hoc test. Results of non-parametric data, including hematocrit, neutrophil, lymphocyte, monocyte, eosinophil and basophil were analyzed by Kruskal-Wallis with Dunn's post hoc test. Different letters (a, b) indicate a statistically significant difference between groups ($p < 0.05$), whereas identical letters indicate no statistically significant difference between groups ($p > 0.05$). WBC, white blood cell count; RBC, red blood cell count; MCV, mean corpuscular volume; MCH, mean corpuscular hematocrit; MCHC, mean corpuscular hemoglobin concentration; PT, prothrombin time.

Table S8. Serum biochemical parameters in male and female Sprague-Dawley rats following 28-day oral administration of LP28.

	Control	Low Dose (500 mg LP28/kg BW)	Medium Dose (1000 mg LP28/kg BW)	High Dose (2000 mg LP28/kg BW)
**Male**				
Glucose (mg /dL)	201.3 ± 41.9 [a]	165.5 ± 53.9 [a]	203.8 ± 41.5 [a]	220.2 ± 27.3 [a]
BUN (mg/dL)	14.5 ± 2.0 [a]	13.0 ± 1.5 [a]	13.7 ± 1.3 [a]	13.5 ± 1.5 [a]
Creatinine (mg/dL)	0.32 ± 0.03 [ab]	0.33 ± 0.03 [b]	0.29 ± 0.02 [a]	0.31 ± 0.02 [ab]
AST (U/L)	82.3 ± 24.7 [a]	65.7 ± 6.2 [a]	81.3 ± 25.4 [a]	70.8 ± 11.7 [a]
ALT (U/L)	32.5 ± 10.4 [a]	26.5 ± 1.9 [a]	27.8 ± 7.1 [a]	32.8 ± 17.7 [a]
Total protein (g/dL)	6.3 ± 0.4 [a]	6.2 ± 0.3 [a]	6.4 ± 0.3 [a]	6.3 ± 0.1 [a]
Albumin (m/dL)	3.5 ± 0.2 [a]	3.5 ± 0.2 [a]	3.5 ± 0.1 [a]	3.4 ± 0.2 [a]
ALP (U/L)	150.3 ± 25.7 [a]	156.2 ± 20.3 [a]	161 ± 36.5 [a]	176.0 ± 17.6 [a]
γ-GT (U/L)[1]	< 3.0	< 3.0	< 3.0	< 3.0
Cholesterol (mg/dL)	58.7 ± 14.8 [a]	60.5 ± 9.3 [a]	58.8 ± 10.3 [a]	52.2 ± 8.6 [a]
Triglyceride (mg/dL)	89.2 ± 27.3 [b]	67.7 ± 29.1 [ab]	75.2 ± 15.9 [ab]	53.2 ± 4.4 [a]
Calcium (mg/dL)	11.2 ± 0.7 [a]	11.6 ± 0.7 [a]	11.9 ± 0.8 [a]	12.1 ± 0.3 [a]
Phosphorus (mg/dL)	13.4 ± 1.5 [a]	13.4 ± 1.3 [a]	13.7 ± 1.6 [a]	13 ± 1.0 [a]
Sodium (meg/L)	143.8 ± 2.3 [a]	147.5 ± 1.2 [a]	145.3 ± 1.2 [a]	147.3 ± 0.5 [a]
Potassium (meg/L)	9.8 ± 1.1 [a]	8.6 ± 1.3 [ab]	9.2 ± 1.2 [ab]	8.0 ± 0.6 [b]
Chloride (meg/L)	96.2 ± 2.0 [a]	97.7 ± 1.0 [a]	96.3 ± 0.8 [a]	96.8 ± 1.5 [a]
Globulin (g/dL)	2.8 ± 0.3 [a]	2.7 ± 0.2 [a]	2.9 ± 0.2 [a]	2.9 ± 0.1 [a]
Total bilirubin (mg/dL)	0.1 ± 0.0 [a]	0.1 ± 0.0 [a]	0.1 ± 0.0 [a]	0.1 ± 0.0 [a]
**Female**				
	Control	Low Dose (500 mg LP28/kg BW)	Medium Dose (1000 mg LP28/kg BW)	High Dose (2000 mg LP28/kg BW)
Glucose (mg /dL)	146.7 ± 33.1 [a]	171.7 ± 32 [a]	160.7 ± 39.4 [a]	165.3 ± 34.2 [a]
BUN (mg/dL)	13.8 ± 1.3 [a]	14.1 ± 2.0 [a]	14.9 ± 1.5 [a]	15.5 ± 1.5 [a]
Creatinine (mg/dL)	0.4 ± 0.0 [a]	0.3 ± 0.0 [a]	0.3 ± 0.0 [a]	0.3 ± 0.0 [a]
AST (U/L)	69.8 ± 17.2 [a]	62 ± 10.6 [a]	67.3 ± 13.0 [a]	59.7 ± 6.9 [a]
ALT (U/L)	22.3 ± 6.6 [a]	19.5 ± 2.6 [a]	22.2 ± 3.7 [a]	17.2 ± 2.9 [a]
Total protein (g/dL)	7.2 ± 0.5 [b]	6.7 ± 0.5 [ab]	6.8 ± 0.3 [ab]	6.4 ± 0.2 [a]
Albumin (m/dL)	4.1 ± 0.3 [b]	3.8 ± 0.3 [ab]	3.9 ± 0.1 [ab]	3.7 ± 0.1 [a]
ALP (U/L)	87 ± 28.5 [a]	68.3 ± 18.7 [a]	81.3 ± 21.9 [a]	84.0 ± 13.3 [a]
γ-GT (U/L)[1]	< 3.0	< 3.0	< 3.0	< 3.0
Cholesterol (mg/dL)	94.5 ± 17.0 [a]	81.0 ± 13.7 [a]	88.7 ± 12.9 [a]	81.7 ± 17.7 [a]
Triglyceride (mg/dL)	52.5 ± 8.1 [a]	54.7 ± 15.9 [a]	56.8 ± 20.2 [a]	53.8 ± 15.2 [a]
Calcium (mg/dL)	11.7 ± 0.3 [a]	11.8 ± 0.4 [a]	12.3 ± 0.5 [a]	12.1 ± 0.5 [a]
Phosphorus (mg/dL)	10.7 ± 1.1 [a]	10.6 ± 0.6 [a]	11.4 ± 0.9 [a]	11.8 ± 0.9 [a]
Sodium (meg/L)	144.2 ± 1.0 [a]	143.7 ± 1.4 [a]	143.3 ± 1.0 [a]	145.2 ± 1.6 [a]
Potassium (meg/L)	8.7 ± 0.9 [ab]	8.6 ± 1.2 [ab]	9.8 ± 0.6 [b]	7.4 ± 0.5 [a]
Chloride (meg/L)	98.0 ± 0.6 [a]	97.5 ± 1.4 [a]	97.3 ± 1.4 [a]	97.5 ± 1.4 [a]
Globulin (g/dL)	3.1 ± 0.2 [a]	2.9 ± 0.2 [a]	2.9 ± 0.2 [a]	2.8 ± 0.2 [a]
Total bilirubin (mg/dL)	0.2 ± 0.1 [a]	0.1 ± 0.0 [a]	0.1 ± 0.1 [a]	0.1 ± 0.0 [a]

Data are shown as mean ± SD, $n = 12$. Statistical significances between the values of each group and negative control were analyzed by one-way ANOVA with a post-hoc Tukey test. Different letters (a, b) indicate a significant difference between groups ($p < 0.05$), whereas identical letters indicate no statistically significant difference between groups ($p > 0.05$). [1] γ-GT shown as lower the detection limit. BUN, blood urea nitrogen; AST, aspartate aminotransferase; ALT, alanine aminotransferase; ALP, alkaline phosphatase.

Table S9. Antimicrobial Resistance genes analysis of LP28.

Database	Gene id	Location	Hit	Known species	E value	Identity (%)	Coverage (%)	Possible Function
CARD	ND							
AMRFinderPlus	ND							
ResFinder	ClpL	3136236–3138350				98.2		Temperature
ARG-ANNOT	ND							

Table S10. List of insertion sequences (IS) identified in the genome of LP28.

No.	IS	IS family	Function	Start	End
1	ISP2	IS1182	Transposase	9246	11041
2	ISP2	IS1182	Transposase	866306	868101
3	ISP2	IS1182	Transposase	1618198	1619993
4	ISP2	IS1182	Transposase	2937737	2939532
5	ISP2	IS1182	Transposase	3155037	3156832
6	ISP1	ISL3	Transposase	302586	304018
7	ISP1	ISL3	Transposase	595999	597431
8	ISP1	ISL3	Transposase	672969	674401
9	ISP1	ISL3	Transposase	1569892	1571324
10	ISP1	ISL3	Transposase	1937081	1938513
11	ISP1	ISL3	Transposase	2027620	2029052
12	ISLsa1	IS30	Transposase	34891	35924
13	ISLsa1	IS30	Transposase	38483	39516
14	ISLpl1	IS30	Transposase	1460	2502
15	ISLpl1	IS30	Transposase	45482	46524
16	ISLpl1	IS30	Transposase	32757	33799

Table S11. Bioinformatic analysis of phage sequences in the genome of LP28.

Region	Region Length	Completeness	Total No. of Proteins	Region Position	Most Common Phage (number of gene hit)
1	53.8 Kb	intact	58	1252287−1306126	PHAGE_Lactob_Sha1_NC_019489(23)
2	44.5 Kb	intact	58	2089197−2133710	PHAGE_Oenoco_phi9805_NC_023559(17)
3	40.9 Kb	intact	50	2827356−2868330	PHAGE_Lactob_Sha1_NC_019489(28)
